# Definitions of pulmonary exacerbation in people with cystic fibrosis: a scoping review

**DOI:** 10.1136/bmjresp-2024-002456

**Published:** 2024-08-14

**Authors:** Maryam Almulhem, Christopher Ward, Iram Haq, Robert D Gray, Malcolm Brodlie

**Affiliations:** 1 Translational and Clinical Research Institute, Newcastle University, Newcastle upon Tyne, UK; 2 College of Applied Medical Sciences, King Faisal University, Al-Ahsa, Saudi Arabia; 3 Paediatric Respiratory Medicine, Great North Children's Hospital, Newcastle upon Tyne Hospitals NHS Foundation Trust, Newcastle upon Tyne, UK; 4 School of Infection and Immunity, University of Glasgow, Glasgow, UK

**Keywords:** pulmonary exacerbation, cystic fibrosis, definition

## Abstract

**Background:**

Pulmonary exacerbations (PExs) are clinically important in people with cystic fibrosis (CF). Multiple definitions have been used for PEx, and this scoping review aimed to identify the different definitions reported in the literature and to ascertain which signs and symptoms are commonly used to define them.

**Methods:**

A search was performed using Embase, MEDLINE, Cochrane Library, Scopus and CINAHL. All publications reporting clinical trials or prospective observational studies involving definitions of PEx in people with CF published in English from January 1990 to December 2022 were included. Data were then extracted for qualitative thematic analysis.

**Results:**

A total of 14 039 records were identified, with 7647 titles and abstracts screened once duplicates were removed, 898 reviewed as full text and 377 meeting the inclusion criteria. Pre-existing definitions were used in 148 publications. In 75% of papers, an objective definition was used, while 25% used a subjective definition, which subcategorised into treatment-based definitions (76%) and those involving clinician judgement (24%). Objective definitions were subcategorised into three groups: those based on a combination of signs and symptoms (50%), those based on a predefined combination of signs and symptoms plus the initiation of acute treatment (47%) and scores involving different clinical features each with a specific weighting (3%). The most common signs and symptoms reported in the definitions were, in order, sputum production, cough, lung function, weight/appetite, dyspnoea, chest X-ray changes, chest sounds, fever, fatigue or lethargy and haemoptysis.

**Conclusion:**

We have identified substantial variation in the definitions of PEx in people with CF reported in the literature. There is a requirement for the development of internationally agreed-upon, standardised and validated age-specific definitions. Such definitions would allow comparison between studies and effective meta-analysis to be performed and are especially important in the highly effective modulator therapy era in CF care.

**WHAT IS ALREADY KNOWN ON THIS TOPIC**

- Pulmonary exacerbations (PExs) are clinically important in cystic fibrosis (CF) and are frequently used as outcome measures in clinical research studies.
- Multiple definitions of PEx exist.

**WHAT THIS STUDY ADDS**

- Substantial variation in the types of definitions used for PEx in people with CF was identified in this scoping review.
- Common themes in these definitions were identified, along with a lack of age-specific definitions.
- This limits the comparison between studies and effective meta-analysis to be performed.

**HOW THIS STUDY MIGHT AFFECT RESEARCH, PRACTICE OR POLICY**

- There is a requirement for the development of internationally agreed-upon, standardised and validated definitions of PEx in CF that are age-specific.
- This is especially pertinent in the era of highly effective CF transmembrane modulator therapies for many people with CF.

## Introduction

 Lung disease remains the major cause of morbidity and mortality in people with cystic fibrosis (CF).^1^ This is characterised by intermittent episodes of increased symptoms known as pulmonary exacerbations (PExs).^2^ PEx are typically managed with antibiotics and intensified chest physiotherapy.^3 4^ If a PEx is severe, then hospitalisation may be required.^5^ Multiple studies have demonstrated the clinical importance of PEx in people with CF. In around a quarter of patients, there is a failure to recover to baseline lung function after a PEx.^6 7^ Furthermore, PEx frequency has been associated with the rate of lung function decline in both adults and children.^8^ Ultimately, people who experience frequent PEx have reduced survival.^9^ PEx also impact quality of life and are associated with a financial burden in terms of healthcare costs and time away from employment.^10 11^

Due to the importance of PEx, they are frequently used as a primary or secondary outcome measure in clinical trials,^12^ for example, trials of recombinant human DNase,^13^ tobramycin,^14^ hypertonic saline,^15^ azithromycin^16^ and CF transmembrane conductance regulator (CFTR) modulators.^17 18^ Despite this, there is no agreed-upon uniform definition of PEx and a lack of age-specific criteria.^12^ This heterogeneity makes it challenging to evaluate, meta-analyse or compare findings between studies and limits meaningful conclusions to impact clinical practice.

We performed a scoping review to summarise the definitions of PEx used in the CF research literature, the various criteria used and identify common themes. A scoping review was felt to be most appropriate to examine the extent, nature, range and variety of evidence on this extensive topic.^19^ These findings will feed into the development of consensus-based age-specific definitions by the European Cystic Fibrosis Society Pulmonary Exacerbation Working Group.

## Methods

### Overview

The Preferred Reporting Items for Systematic reviews and Meta-Analyses extension for Scoping Reviews (PRISMA-ScR) checklist was used to report our findings.^20^ The research question was developed using the ‘PICo tool,’ where P stands for population, I for interest and Co for context.^21^ We formulated the following research question: ‘What definitions are reported for PEx in people of different age groups with CF in primary research studies?’. Alongside this, we also asked: ‘What are the most common signs and symptoms reported in definitions of PEx in people with CF?’.

### Criteria for including publications

To get a comprehensive understanding of the definitions used in primary research studies, all clinical trials (randomised clinical trials (RCTs) and non-RCTs) and prospective observational studies that reported PEx as an outcome were included. Publications had to be available in English and clearly specify a PEx definition used in people with CF of any age group. Studies published in abstract form only were considered. Authors were contacted by email if publications were not accessible. Review articles, retrospective studies, case reports and in vitro and in vivo experimental studies were excluded.

### Search strategy

Embase, MEDLINE, Cochrane Library, Scopus and CINAHL databases were searched between January 1990 and December 2022. A comprehensive search strategy was developed in consultation with a librarian specialising in health databases. Medical subject headings and keywords were used with ‘AND’ and ‘OR’ to narrow or broaden the search, depending on the search strategy for each database. Specific details outlining the search strategies for each database can be found in the online supplemental material. The following search terms were employed: ‘cystic fibrosis’ AND ‘pulmonary exacerbation’, limited to English language and 1990–2022. Several pilot searches were run in each database to ensure that key articles were identified. All the references were then imported into Endnote 20 reference management software (Clarivate, London, UK). Duplication was checked using different field settings, as suggested by Bramer *et al*.^22^ After removing the duplicates, all results were exported to the Rayyan website^23^ to screen the title and abstract.

### Study selection and data charting

The titles and/or abstracts of studies retrieved were screened by the primary reviewer (MA). Decisions about the inclusion or exclusion of studies were made between two reviewers (MA and MB). Eligible studies were then retrieved and assessed in full by the primary reviewer (MA). A second reviewer (MB) screened 10% of the included articles to ensure accuracy and agreement. An electronic data recording form was designed for the specific methodology used in the review. The form was piloted on three articles and subsequently optimised.

### Data synthesis

A thematic qualitative synthesis was used for this study^24^ to facilitate the systematic identification of prominent themes and summarise definitions under these themes in a structured way. Thematic synthesis has three stages: the coding of text line by line, the development of descriptive themes and the generation of analytical themes. The charted data were pooled and then coded. Similarities and differences between codes were identified to begin grouping them.^25^ Two main themes were developed from the coded data based on clinical presentation and treatment. For each theme, the frequencies of extracted definitions were reported along with the signs and symptoms used.

### Patient and public involvement

There was no specific patient or public involvement in this scoping review.

## Results

### Characteristics of articles identified by the search strategy

**Figure 1:**
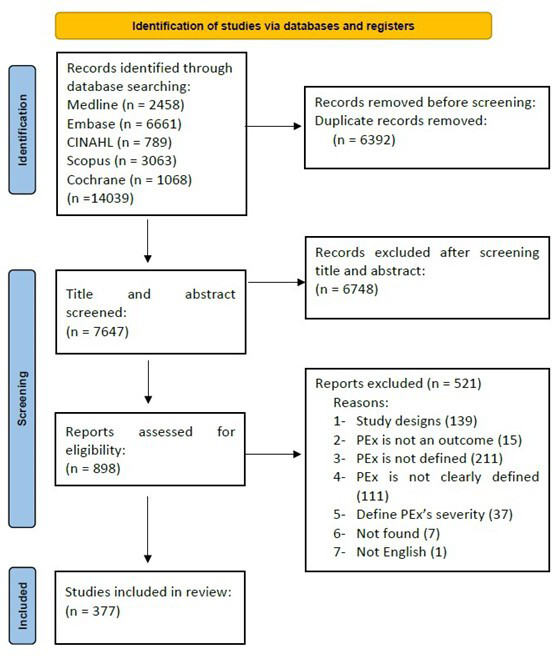
Preferred Reporting Items for Systematic reviews and Meta-Analyses flow diagram.PEx, pulmonary exacerbation.

In total, 14 039 publications were identified by the search strategy across all databases. The PRISMA flow diagram for the study is shown in figure 1. From these, 377 articles met the inclusion criteria for the scoping review and were included and analysed in the qualitative synthesis (the full list of included articles is provided in the online supplemental material). These consisted of 272 observational studies and 105 experimental studies. Most studies were full articles, while 16 studies were available in abstract form only.

### Themes of definitions used for PEx

**Figure 2:**
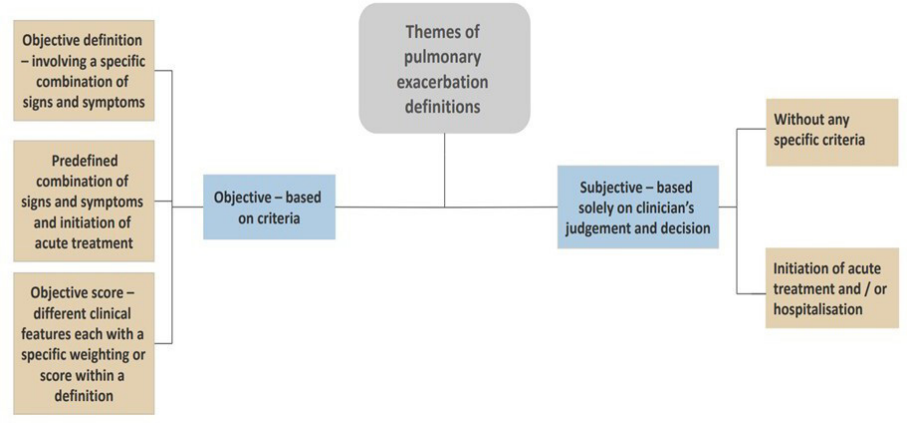
Pulmonary exacerbation definitions and themes.

**Figure 3:**
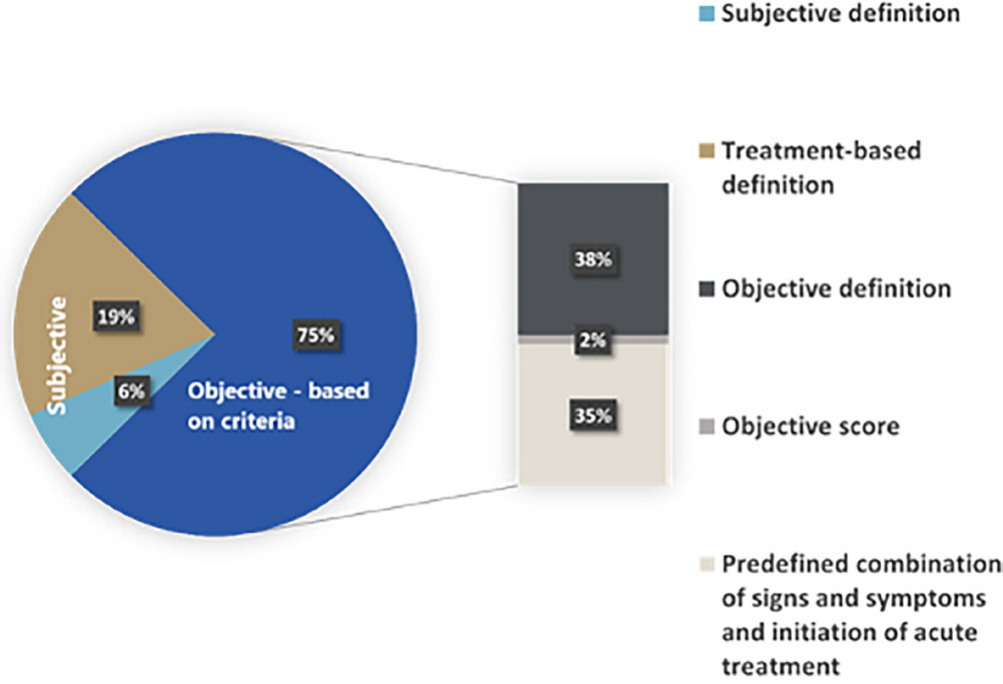
Frequency of pulmonary exacerbation definitions and themes reported in studies.

Two key themes were developed for PEx definitions used in the included articles. After manually coding the definitions, the definitions of PEx were categorised into two themes: those based on objective criteria and those using subjective criteria, as per figure 2. The breakdown of studies: 94 (25%) studies used a subjective definition and 283 (75%) articles used an objective definition as shown in figure 3. The duration of signs and symptoms or length of the treatment course, was included in 23 publications. The duration of signs and symptoms varied from 3 days (nine studies), 5 days (four studies) or 7 days (five studies). Others delineated a minimum course of treatment of 7 days to meet the criteria of PEx (three studies), whereas others required the administration of an antibiotic course for 21 days (two studies).

### Commonly used definitions

Out of the 377 articles reviewed, 148 studies used previously established definitions. There were six commonly used definitions that were identified from the analysis of the articles, as shown in table 1. Full details of these definitions are included in the online supplemental material.

**Table 1:** Commonly used definitions for pulmonary exacerbation

Definition	Number of publications (%)
Fuchs modifications	73 (49%)
Fuchs definition (1994)	38 (26%)
Cystic Fibrosis Foundation (1994)	22 (15%)
Early Pseudomonas Infection Control study (2009)	12 (8%)
Akron Pulmonary Exacerbation Score (2009)	4 (3%)
Rosenfeld definition (2001)	4 (3%)

#### Fuchs definition (1994)

The most commonly used definition was originally published by Fuchs *et al*^13^ in a trial of nebulised recombinant human DNase. The Fuchs definition for PEx is based on a combination of signs and symptoms (at least 4 out of 12 possible) and the need for intravenous antibiotics in people with CF aged >5 years. Various modifications of the original Fuchs definition have been developed and used in studies. Typically, these involve an alteration of the criteria for intravenous treatment or variations in the signs and symptoms required.

These include:

- The definition proposed by the EuroCareCF Working Group in 2011.^26^
- The ‘expanded Fuchs criteria/definition’ broadens the definition to any oral, inhaled and/or intravenous antibiotics prescribed in response to the presence of 4 out of the 12 Fuchs criteria.^18^
- The ‘modified Fuchs criteria’ involve only the presence of 4 out of 12 signs and symptoms without a specified requirement for treatment.

These subtly different definitions have led to some inconsistency in their use and meaning in the literature. For example, some studies use ‘modified Fuchs’ for the EuroCareCF Working Group definition,^26^ while others use ‘modified Fuchs’ to mean the criteria described above, where there is no requirement for intravenous antibiotic treatment.^27 28^ Another modified Fuchs definition excludes one of the original 12 signs/symptoms.^10^ Due to this variation in terminology, some articles were excluded from this review if they did not specify clearly which Fuchs definition was used (original, modified or expanded).^29 30^

#### US CF Foundation (CFF) definition

In 2005, the CFF Microbiology and Infectious Disease Consensus defined a PEx as 3 out of 11 signs or symptoms being present. There was no specification of the age of the patient in the original definition. Studies have used this definition in paediatric patients aged 2–12 years,^31^ although it requires a measurement of forced expiratory volume in 1 s usually done for 6 years old and above only.

#### The Early Pseudomonas Infection Control (EPIC) definition

The EPIC definition published by Treggiari *et al* in 2009 is based on signs and symptoms and specifically applies to children with CF aged 1–12 years.^32^ It divides signs and symptoms into four major and six minor criteria. A presentation involving one major and/or two of the minor criteria signs or symptoms for a specified duration fulfils the definition of a PEx. Some studies have also used the EPIC definition in adults and children aged over 12 years.^33 34^ Another adjustment to the EPIC definition in some studies is to shorten the duration of symptoms for the minor criteria from 5 to 3 days.^35 36^

#### Akron Pulmonary Exacerbation Score (PES) definition

An alternative method to objectively define a PEx is to use a scoring system where each sign or symptom has a specific weighting, with a total score greater than a predefined value being diagnostic of a PEx. The Akron PES^37^ was designed for people with CF aged over 6 years. This score contains 14 elements that are divided into systemic, pulmonary signs and symptoms, and objective measurements. A score of 5 or greater is required to meet the criteria for a PEx. Although the original PES definition was developed for a population aged over 6 years, it has been used in studies for participants down to the age of 6 months, for example, by Hoen *et al*.^38^

#### Rosenfeld definition

The Rosenfeld definition^39^ is another example of a score-based method that involves two models. This score is designed for use in a population aged 6 years and above. It was used in four publications that we identified.

### Definitions used in different age groups

**Figure 4:**
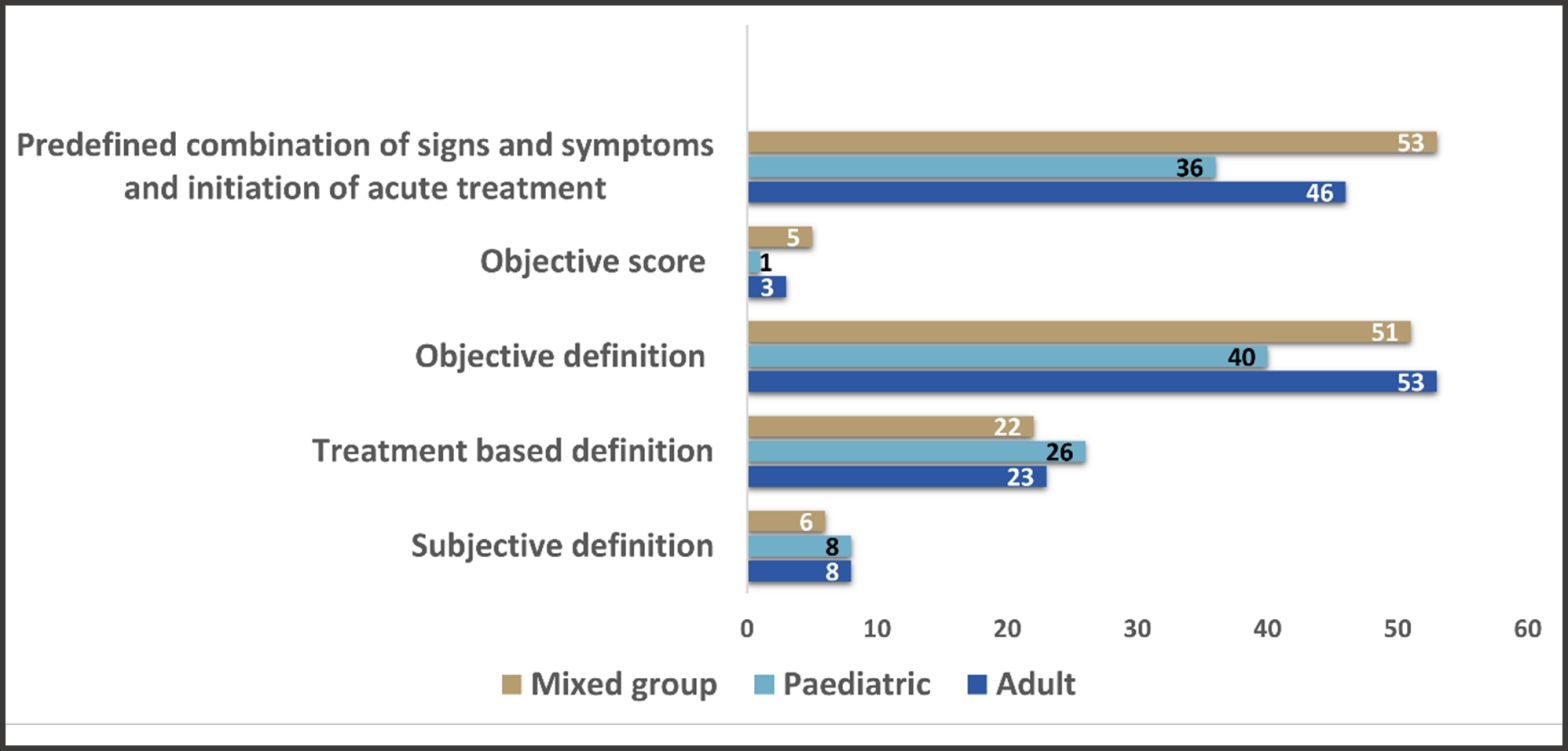
Types of definitions reported in paediatric and adult studies.

Out of a total of 377 articles, only one of the included abstracts did not report participant age clearly. We subsequently divided these populations into three groups: adult (aged ≥18 years), paediatric (<18 years) and mixed for studies applying definitions to both age groups. We found that in the specific paediatric and adult groups, an objective definition, defining PEx based on signs and symptoms, was the most commonly used. However, a predefined combination of signs and symptoms and the initiation of an acute treatment definition were used most often in studies with a mixed age group (figure 4). A breakdown of the definitions used in studies involving the different age groups is provided in table 2.

**Table 2:** Specific definitions used by studies involving different age groups

Definition	Adults	Paediatrics	Mixed
Fuchs definition (1994)	16	8	14
Fuchs modifications	23	22	28
Cystic Fibrosis Foundation (1994)	7	4	11
Early Pseudomonas Infection Control study (2009)	0	6	6
Akron Pulmonary Exacerbation Score	2	0	2
Rosenfeld definition (2001)	1	1	2

### Commonly used signs and symptoms in PEx definitions

**Figure 5:**
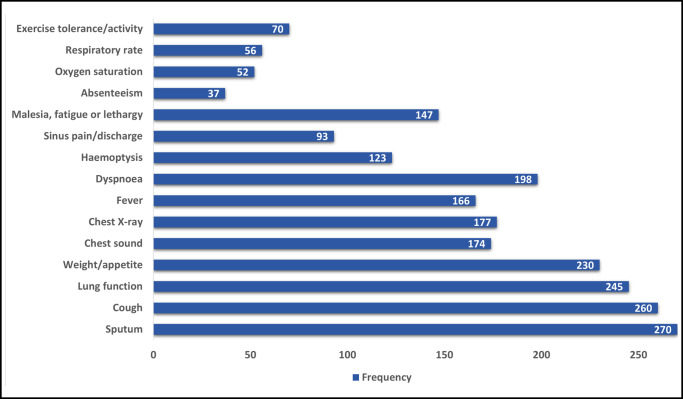
Most common signs and symptoms used in pulmonary exacerbation definitions.

The second aim of this review was to examine the signs and symptoms used in the different definitions of PEx. Out of the 377 included publications, 288 defined PEx using signs and symptoms. The frequency of these different signs and symptoms is shown in figure 5. Common signs and symptoms (in 75% of included studies) used in defining PEx were mainly respiratory signs and symptoms such as an increase in the amount of sputum, increased cough and a decline in lung function, followed by systemic features such as weight loss and decreased appetite.

## Discussion

The results of this scoping review highlight large amounts of variation in definitions reported and used for PEx in CF primary research literature. Some studies proposed their own novel definition of a PEx, with the remainder instead using definitions or elements derived from previous studies. This heterogeneity and lack of an accepted and validated standardised definition make it challenging to design clinical trials with PEx as a clinical outcome and limit meta-analysis and comparison of studies. It may be argued that the lack of standardised definitions reduces the effectiveness and applicability of clinical research in CF at present.

Using a predefined combination of signs and symptoms is the most frequently used method to define PEx. This approach has some objectivity and depends on clinical presentation and parameters rather than clinical interventions. However, there may still be inconsistencies in the symptoms and their duration used to define PEx. Another factor that should be considered is the difference in clinical presentations between adults and children when determining which clinical characteristics best define a PEx. The difference in reported signs and symptoms in different age groups is illustrated by various studies in this review.^40^,^42^ Arguably, there is no single definition that will fit all age groups, and criteria must be age specific to be truly clinically relevant. Adult and paediatric populations vary in their ability to perform spirometry reliably, typical presenting symptoms^40 41^ and the preferred route of treatment.^43^

The need for a change in treatment, most commonly prescribing antibiotics, is frequently part of the definitions used for PEx.^44 45^ Few would disagree that this is a crucial intervention relevant to both patients and multidisciplinary clinical teams. However, there is variation in the threshold for recognising and treating PEx at multiple levels in reality.^46^ Indications for oral, inhaled or intravenous antibiotics also vary between centres and individual clinicians. For these reasons, the use of treatment-based definitions was not recommended by the EuroCareCF Group^26^ and CFF,^47^ arguing that treatment as a definition of PEx in CF should not stand alone because of rapid changes in treatment modalities. As the rate of intravenous antibiotic administration at home has increased, using this definition will not capture such cases.^48^ Despite this, predefined signs and symptoms and treatment are widely employed in the included articles (35%), while a definition based on treatment or hospitalisation is less prevalent (19%).

The least well-specified method to define a PEx is simply a subjective judgement made by the attending clinician. This method was used in nine publications included in this review. This type of definition is open to variability and bias depending on physicians’ preferences and backgrounds. Such a definition is ineffective as a clinical outcome in clinical trials. Two scoring systems to define PEx were found in the included literature. These scores were based on clinical assessment and were independent of treatment decisions.^37 39^ The advantage of these scoring methods is that they do not rely on therapeutic decisions as recommended by the CFF Consensus Conference on Outcome Measures for Clinical Trials^47^ and the EuroCareCF Group^26^ because of rapid developments in treatment modalities.

Rather than definitions based on clinical presentation and/or treatment, patient-reported indicators of PEx have been suggested to help recognise PEx at an earlier stage and facilitate prompt intervention.^40 41^ Abbott and colleagues^40^ interviewed adults with CF who experienced exacerbations. They asked them to report symptoms experienced during PEx and how they consequently recognised when these had resolved.^40^ For many patients, the onset of an exacerbation was recognised by fatigue and alterations in sleep, cough, sputum, appetite, mood and daily activities. When describing the improvement, they reported enhancement mainly in the activity level, ability to sleep, cough and less sputum production. Abbott and partners extended their work to include children, using the same method.^41^ They found that, in general, the most frequently reported symptoms for the onset of exacerbations were tiredness and increased cough and ‘cold’ symptoms, while in moderate or severe disease, activity-induced breathlessness, sleep disturbances and mood fluctuations were most common.^41^ Those with severe disease also reported increases in sputum production and lack of appetite.^41^ The child-reported indicators of PEx tended to map onto those reported by adults, with some exceptions.^41^ Although these findings support the concept that there are similarities between reported indicators of PEx across the CF lifespan, developing tools to monitor the progression of the disease, requisite intervention and progression of treatment that are applicable to all ages is likely to be challenging.^49^

This scoping review’s strengths involve the comprehensive search strategy of five large and reliable databases (Embase, MEDLINE, Cochrane Library, Scopus and CINAHL) and seek to summarise the entire range of PEx definitions reported in the primary research CF literature. The review covers a long period from 1990 to 2022, and we performed an extensive review of the reference lists from relevant studies to avoid missing other potentially important papers. However, the review was limited to studies in English only, potentially missing some relevant non-English papers.

## Conclusions

This scoping review affirms that there is no single unified definition of PEx in people with CF used in the research literature. Rather, several unvalidated definitions are commonly used, with considerable variation between the different definitions. This inconsistency in defining a PEx is almost certainly detrimental to clinical research by making comparisons of studies challenging and limiting meta-analysis. There is a need for internationally agreed-upon and age-specific definitions of PEx that include specific signs and symptoms and the need for treatment. Moving forward, definitions should reflect the impact of highly effective CFTR modulators on the clinical characteristics of PEx in people with CF who are eligible.^50^ We suggest that consensus-based definitions for PEx could be reached by integrating the criteria of the PEx definitions found in this review together with the opinion of multidisciplinary experts, practising healthcare professionals and people with CF themselves.

## supplementary material

10.1136/bmjresp-2024-002456 online supplemental file 1

## Data Availability

All data relevant to the study are included in the article or uploaded as supplementary information.
