# Supplementary material for: Definitions of pulmonary exacerbation in people with cystic fibrosis: a scoping review
Source: BMJ Open Respir Res. 2024 Aug 14;11(1):e002456. doi: 10.1136/bmjresp-2024-002456 (PMC11331921; doi:10.1136/bmjresp-2024-002456)
Supplement: online supplemental file 1 [file bmjresp-11-1-s001.pdf]

SUPPLEMENTARY MATERIAL

DEFINITIONS OF PULMONARY EXACERBATION IN PEOPLE WITH CYSTIC FIBROSIS: A SCOPING REVIEW

Maryam M Amulhem[1,2], Christopher Ward[1], Iram Haq[1,3] Robert D Gray[4] and Malcolm Brodlie[1,3]*

[1]Translational and Clinical Research Institute, Faculty of Medical Sciences, Newcastle University, Newcastle upon Tyne, United Kingdom.

[2] College of Applied Medical Sciences, King Faisal University, Al-Ahsa, Saudi Arabia.

[3]Paediatric Respiratory Medicine, Great North Children's Hospital, Newcastle upon Tyne Hospitals NHS Foundation Trust, Newcastle upon Tyne, UK Queen Victoria Road, Newcastle upon Tyne, United Kingdom.

[4]School of Infection and Immunity, University of Glasgow, Glasgow, United Kingdom.

Full list of included articles

Citation (Harvard at Newcastle style)
1. Aaron, S. D., Vandemheen, K. L., Ferris, W., Fergusson, D., Tullis, E., Haase, D., Berthiaume, Y., Brown, N., Wilcox, P., Yozghatlian, V., Bye, P., Bell, S., Chan, F., Rose, B., Jeanneret, A., Stephenson, A., Noseworthy, M., Freitag, A., Paterson, N. and Doucette, S. (2005) 'Combination antibiotic susceptibility testing to treat exacerbations of cystic fibrosis associated with multiresistant bacteria: a randomised, double-blind, controlled clinical trial', Lancet, 366(9484), pp. 463-471.
2. Abbott, J., Holt, A., Hart, A., Morton, A. M., MacDougall, L., Pogson, M., Milne, G., Rodgers, H. C. and Conway, S. P. (2009) 'What defines a pulmonary exacerbation? The perceptions of adults with cystic fibrosis', Journal of cystic fibrosis : official journal of the European Cystic Fibrosis Society, 8(5), pp. 356-9.
3. Abbott, J., Holt, A., Morton, A. M., Hart, A., Milne, G., Wolfe, S. P. and Conway, S. P. (2012) 'Patient indicators of a pulmonary exacerbation: preliminary reports from school aged children map onto those of adults', Journal of cystic fibrosis : official journal of the European Cystic Fibrosis Society, 11(3), pp. 180-6.
4. Accurso, F. J., Moss, R. B., Wilmott, R. W., Anbar, R. D., Schaberg, A. E., Durham, T. A. and Ramsey, B. W. (2011) 'Denufosol tetrasodium in patients with cystic fibrosis and normal to mildly impaired lung function', American Journal of Respiratory & Critical Care Medicine, 183(5), pp. 627-634.
5. Al Ansari, N. A., Foweraker, J., Mackeown, D. and Bilton, D. (2006) 'Evaluation of once daily tobramycin versus the traditional three time daily for the treatment of acute pulmonary exacerbations in adult cystic fibrosis patients', Qatar Medical Journal, 15(1), pp. 34-38.
6. Alison, J. A., Donnelly, P. M., Lennon, M., Parker, S., Torzillo, P., Mellis, C. and Bye, P. T. (1994) 'The effect of a comprehensive, intensive inpatient treatment program on lung function and exercise capacity in patients with cystic fibrosis', Physical Therapy, 74(6), pp. 583-3.
7. Amelina, E. L., Krasovsky, S. A., Akhtyamova-Givirovskaya, N. E., Kashirskaya, N. Y., Abdulganieva, D. I., Asherova, I. K., Zilber, I. E., Kozyreva, L. S., Kudelya, L. M., Ponomareva, N. D., Revel-Muroz, N. P., Reutskaya, E. M., Stepanenko, T. A., Seitova, G. N., Ukhanova, O. P., Magnitskaya, O. V., Kudlay, D. A., Markova, O. A. and Gapchenko, E. V. (2021) 'Comparison of biosimilar Tigerase and Pulmozyme in long-term symptomatic therapy of patients with cystic fibrosis and severe pulmonary impairment (subgroup analysis of a Phase III randomized open-label clinical trial (NCT04468100))', PloS one, 16(12), pp. e0261410.
8. Aquino, C. S. B. d., Rodrigues, J. C. and Silva-Filho, L. V. R. F. d. (2022) 'Routine spirometry in cystic fibrosis patients: impact on pulmonary exacerbation diagnosis and FEV1 decline', Jornal brasileiro de pneumologia : publicacao oficial da Sociedade Brasileira de Pneumologia e Tisilogia, 48(3), pp. e20210237.
9. Baker, E. H., Burgess, J. C., Bilton, D., Hodson, M. E., Gyi, K. M. and Srivastava, S. A. (2010) 'Comparison of cystic fibrosis exacerbations requiring oral or intravenous antibiotics', Pediatric pulmonology, 45(SUPPL. 33), pp. 376-377.
10. Balfour-Lynn, I. M., Lees, B., Hall, P., Phillips, G., Khan, M., Flather, M. and Elborn, J. S. (2006) 'Multicenter randomized controlled trial of withdrawal of inhaled corticosteroids in cystic fibrosis', American Journal of Respiratory & Critical Care Medicine, 173(12), pp. 1356-1362.
11. Baran, E., Butti, F., Hendriksen, B., Granero, N., D'Ascenzo, V., Pistorio, V., Volta, L., Menna, L. and Garcia, G. (2016) 'Body fat percentage is a risk factor for exacerbations in adults with cystic fibrosis', Journal of Cystic Fibrosis, 15(Supplement 1), pp. S102.
12. Benlala, I., Hocke, F., Macey, J., Bui, S., Berger, P., Laurent, F. and Dournes, G. (2020) 'Quantification of MRI T2-weighted High Signal Volume in Cystic Fibrosis: A Pilot Study', Radiology, 294(1), pp. 186-196.
13. Benlala, I., Point, S., Leung, C., Berger, P., Woods, J. C., Raherison, C., Laurent, F., Macey, J. and Dournes, G. (2020) 'Volumetric quantification of lung MR signal intensities using ultrashort TE as an automated score in cystic fibrosis', European Radiology, 30(10), pp. 5479-5488.
14. Bilton, D., Robinson, P., Cooper, P., Gallagher, C. G., Kolbe, J., Fox, H., Jaques, A. and Charlton, B. (2011) 'Inhaled dry powder mannitol in cystic fibrosis: an efficacy and safety study', The European respiratory journal, 38(5), pp. 1071-80.

15. Block, J. K., Vandemheen, K. L., Tullis, E., Fergusson, D., Doucette, S., Haase, D., Berthiaume, Y., Brown, N., Wilcox, P., Bye, P., Bell, S., Noseworthy, M., Pedder, L., Freitag, A., Paterson, N. and Aaron, S. D. (2006) 'Predictors of pulmonary exacerbations in patients with cystic fibrosis infected with multi-resistant bacteria', Thorax, 61(11), pp. 969-74.

16. Bouka, A., Tiede, H., Liebich, L., Dumitrascu, R., Hecker, C., Reichenberger, F., Mayer, K., Seeger, W. and Schulz, R. (2012) 'Quality of life in clinically stable adult cystic fibrosis out-patients: associations with daytime sleepiness and sleep quality', Respiratory medicine, 106(9), pp. 1244-9.

17. Bradley, J., Howard, J., Wallace, E., Elborn, S., Bradley, J., Howard, J., Wallace, E. and Elborn, S. (2000) 'Reliability, repeatability, and sensitivity of the modified shuttle test in adult cystic fibrosis', CHEST, 117(6), pp. 1666-1671.

18. Bradley, J. M., Blume, S. W., Balp, M.-M., Honeybourne, D. and Elborn, J. S. (2013) 'Quality of life and healthcare utilisation in cystic fibrosis: a multicentre study', The European respiratory journal, 41(3), pp. 571-7.

19. Britto, M. T., Kotagal, U. R., Hornung, R. W., Atherton, H. D., Tsevat, J., Wilmott, R. W., Britto, M. T., Kotagal, U. R., Hornung, R. W., Atherton, H. D., Tsevat, J. and Wilmott, R. W. (2002) 'Impact of recent pulmonary exacerbations on quality of life in patients with cystic fibrosis', CHEST, 121(1), pp. 64-72.

20. Brody, A. S., Sucharew, H., Campbell, J. D., Millard, S. P., Molina, P. L., Klein, J. S., Quan, J., Brody, A. S., Sucharew, H., Campbell, J. D., Millard, S. P., Molina, P. L., Klein, J. S. and Quan, J. (2005) 'Computed tomography correlates with pulmonary exacerbations in children with cystic fibrosis', American Journal of Respiratory & Critical Care Medicine, 172(9), pp. 1128-1132.

21. Bruzzese, E., Raia, V., Ruberto, E., Scotto, R., Giannattasio, A., Bruzzese, D., Cavicchi, M. C., Francalanci, M., Colombo, C., Faelli, N., Dacco, V., Magazzu, G., Costa, S., Lucidi, V., Majo, F. and Guarino, A. (2018) 'Lack of efficacy of Lactobacillus GG in reducing pulmonary exacerbations and hospital admissions in children with cystic fibrosis: A randomised placebo controlled trial', Journal of cystic fibrosis : official journal of the European Cystic Fibrosis Society, 17(3), pp. 375-382.

22. Bruzzese, E., Raia, V., Spagnuolo, M. I., Volpicelli, M., De Marco, G., Maiuri, L. and Guarino, A. (2007) 'Effect of Lactobacillus GG supplementation on pulmonary exacerbations in patients with cystic fibrosis: a pilot study', Clinical Nutrition, 26(3), pp. 322-328.

23. Button, B. M., Wilson, L. M., Burge, A. T., Kimmel, L., Finlayson, F., Williams, E., Talbot, A., Tierney, A., King, S., Holland, A. E., Keating, D., Kotsimbos, T. and Wilson, J. W. (2021) 'The AWESCORE, a patient-reported outcome measure: Development, feasibility, reliability, validity and responsiveness for adults with cystic fibrosis', ERJ Open Research, 7(3), pp. 00120-2021.

24. Byrnes, C. A., Vidmar, S., Cheney, J. L., Carlin, J. B., Armstrong, D. S., Cooper, P. J., Grimwood, K., Moodie, M., Robertson, C. F., Rosenfeld, M., Tiddens, H. A. and Wainwright, C. E. (2013) 'Prospective evaluation of respiratory exacerbations in children with cystic fibrosis from newborn screening to 5 years of age', Thorax, 68(7), pp. 643-651.

25. Carnovale, V., Iacotucci, P., Qiao, D., Ferrillo, L., Somma, J., Buonaurio, S., Marcella, d. I., Celardo, A. and Savi, D. (2022) 'Clinical outcomes of digital health in adults with cystic fibrosis', Respiratory medicine, 202, pp. 106970.

26. Carpio, C., Lerin, M., Torres, I., Fernandez-Velilla, M., Garcia Rio, F., Alvarez-Sala, R. and Prados, C. (2022) 'Factors predicting 6-min walking test indexes in adults with cystic fibrosis', Science and Sports, 37(5-6), pp. 438-445.

27. Carson, S. W., Psoter, K., Koehler, K., Siklosi, K. R., Montemayor, K., Toporek, A., West, N. E., Lechtzin, N., Hansel, N. N., Collaco, J. M. and Merlo, C. A. (2022) 'Indoor air pollution exposure is associated with greater morbidity in cystic fibrosis', Journal of cystic fibrosis : official journal of the European Cystic Fibrosis Society, 21(2), pp. e129-e135.

28. Choyce, J., Shaw, K. L., Sitch, A. J., Mistry, H., Whitehouse, J. L. and Nash, E. F. (2017) 'A prospective pilot study of home monitoring in adults with cystic fibrosis (HOME-CF): protocol for a randomised controlled trial', BMC Pulmonary Medicine, 17, pp. 1-7.

29. Ciet, P., Bertolo, S., Ros, M., Andrinopoulou, E. R., Tavano, V., Lucca, F., Feiweier, T., Krestin, G. P., Tiddens, H. A. W. M. and Morana, G. (2017) 'Detection and monitoring of lung inflammation in cystic fibrosis during respiratory tract exacerbation using diffusion-weighted magnetic resonance imaging', The European respiratory journal, 50(1).

30. Clayton, R. G., Sr., Diaz, C. E., Bashir, N. S., Panitch, H. B., Schidlow, D. V. and Allen, J. L. (1998) 'Pulmonary function in hospitalized infants and toddlers with cystic fibrosis', The Journal of pediatrics, 132(3 Pt 1), pp. 405-8.
31. Cohen-Cymberknoh, M., Ben Meir, E., Gartner, S., Reiter, J., Spangenberg, A., Garriga, L., Eisenstadt, I., Israeli, T., Tsabari, R., Shoseyov, D., Gileles-Hillel, A., Breuer, O., Simanovsky, N. and Kerem, E. (2021) 'How abnormal is the normal? Clinical characteristics of CF patients with normal FEV1', Pediatric pulmonology, 56(7), pp. 2007-2013.
32. Couch, M. J., Munidasa, S., Rayment, J. H., Voskrebenzev, A., Seethamraju, R. T., Vogel-Claussen, J., Ratjen, F. and Santyr, G. (2021) 'Comparison of Functional Free-Breathing Pulmonary 1H and Hyperpolarized 129Xe Magnetic Resonance Imaging in Pediatric Cystic Fibrosis', Academic Radiology, 28(8), pp. e209-e218.
33. da Silva Santos, C. I., de Oliveira Ribeiro, M. A. G., Morcillo, A. M., Ribeiro, A. F. and Ribeiro, J. D. (2010) 'Antibiotic therapy and Effects of Respiratory Physiotherapy Techniques Cystic Fibrosis Patients Treated for Acute Lung Exacerbation: an Experimental Study', Archivos de bronconeumologia, 46(6), pp. 310-316.
34. Davies, J. C., Wainwright, C. E., Canny, G. J., Chilvers, M. A., Howenstine, M. S., Munck, A., Mainz, J. G., Rodriguez, S., Li, H., Yen, K., Ordoñez, C. L. and Ahrens, R. (2013) 'Efficacy and safety of ivacaftor in patients aged 6 to 11 years with cystic fibrosis with a G551D mutation', American Journal of Respiratory & Critical Care Medicine, 187(11), pp. 1219-1225.
35. Davis, S. D., Fordham, L. A., Brody, A. S., Noah, T. L., Retsch-Bogart, G. Z., Qaqish, B. F., Yankaskas, B. C., Johnson, R. C., Leigh, M. W., Davis, S. D., Fordham, L. A., Brody, A. S., Noah, T. L., Retsch-Bogart, G. Z., Qaqish, B. F., Yankaskas, B. C., Johnson, R. C. and Leigh, M. W. (2007) 'Computed tomography reflects lower airway inflammation and tracks changes in early cystic fibrosis', American Journal of Respiratory & Critical Care Medicine, 175(9), pp. 943-950.
36. Dentice, R. L., Elkins, M. R., Middleton, P. G., Bishop, J. R., Wark, P. A. B., Dorahy, D. J., Harmer, C. J., Honghua, H., Bye, P. T. P. and Hu, H. (2016) 'A randomised trial of hypertonic saline during hospitalisation for exacerbation of cystic fibrosis', Thorax, 71(2), pp. 141-147.
37. Dimango, E., Walker, P., Keating, C., Berdella, M., Robinson, N., Langfelder-Schwind, E., Levy, D. and Liu, X. (2014) 'Effect of esomeprazole versus placebo on pulmonary exacerbations in cystic fibrosis', BMC Pulmonary Medicine, 14(1), pp. 21-21.
38. Dobbin, C. J., Bartlett, D., Melehan, K., Grunstein, R. R., Bye, P. T. P., Dobbin, C. J., Bartlett, D., Melehan, K., Grunstein, R. R. and Bye, P. T. P. (2005) 'The effect of infective exacerbations on sleep and neurobehavioral function in cystic fibrosis', American Journal of Respiratory & Critical Care Medicine, 172(1), pp. 99-104.
39. Donadio, M. V. F., Heinzmann-Filho, J. P., Vendrusculo, F. M., Frasson, P. X. H. and Marostica, P. J. C. (2017) 'Six-Minute Walk Test Results Predict Risk of Hospitalization for Youths with Cystic Fibrosis: A 5-Year Follow-Up Study', Journal of Pediatrics, 182, pp. 204-209.e1.
40. Donadio, M. V. F., Vendrusculo, F. M., Campos, N. E., Becker, N. A., de Almeida, I. S., Queiroz, K. C. V., Leite, L. R. and Aquino, E. S. (2021) 'The modified shuttle test as a predictor of risk for hospitalization in youths with cystic fibrosis: A two-year follow-up study: Modified shuttle test as a predictor of hospitalization', Journal of Cystic Fibrosis, 20(4), pp. 648-654.
41. Dwyer, T. J., Robbins, L., Kelly, P., Piper, A. J., Bell, S. C. and Bye, P. T. P. (2015) 'Non-invasive ventilation used as an adjunct to airway clearance treatments improves lung function during an acute exacerbation of cystic fibrosis: a randomised trial', Journal of Physiotherapy (Elsevier), 61(3), pp. 142-147.
42. Eber, E., Trawinska-Bartnicka, M., Sands, D., Bellon, G., Mellies, U., Bolbás, K., Quattrucci, S., Mazurek, H., Widmann, R., Schoergenhofer, C., Jilma, B. and Ratjen, F. (2021) 'Aerosolized lancovutide in adolescents (≥12 years) and adults with cystic fibrosis – a randomized trial', Journal of Cystic Fibrosis, 20(1), pp. 61-67.
43. Elborn, J. S., Konstan, M. W., Taylor-Cousar, J. L., Fajac, I., Horsley, A., Sutharsan, S., Aaron, S. D., Daines, C. L., Uluer, A., Downey, D. G., Lucidi, V. V., Ahuja, S., Springman, E., Mershon, J., Grosswald, R. and Rowe, S. M. (2021) 'Empire-CF study: A phase 2 clinical trial of leukotriene A4 hydrolase inhibitor acebilustat in adult subjects with cystic fibrosis', Journal of cystic fibrosis : official journal of the European Cystic Fibrosis Society, 20(6), pp. 1026-1034.
44. Elkins, M. R., Robinson, M., Rose, B. R., Harbour, C., Moriarty, C. P., Marks, G. B., Belousova, E. G., Xuan, W. and Bye, P. T. P. (2006) 'A controlled trial of long-term inhaled hypertonic saline in patients with cystic fibrosis', The New England journal of medicine, 354(3), pp. 229-40.

45. Fathi, H., Moon, T., Donaldson, J., Jackson, W., Sedman, P. and Morice, A. H. (2009) 'Cough in adult cystic fibrosis: Diagnosis and response to fundoplication', Cough, 5(1).

46. Flume, P. A., VanDevanter, D. R., Morgan, E. E., Dudley, M. N., Loutit, J. S., Bell, S. C., Kerem, E., Fischer, R., Smyth, A. R., Aaron, S. D., Conrad, D., Geller, D. E. and Elborn, J. S. (2016) 'A phase 3, multi-center, multinational, randomized, double-blind, placebo-controlled study to evaluate the efficacy and safety of levofloxacin inhalation solution (APT-1026) in stable cystic fibrosis patients', Journal of cystic fibrosis : official journal of the European Cystic Fibrosis Society, 15(4), pp. 495-502.

47. Foong, R. E., Ramsey, K. A., Harper, A. J., Turkovic, L., Rosenow, T., Skoric, B., King, L., Davis, M., Clem, C., Stick, S. M., Davis, S. D., Ranganathan, S. and Hall, G. (2018) 'The utility of lung clearance index to monitor early lung disease in young children with cystic fibrosis', Pediatric pulmonology, 53(Supplement 2), pp. 243-244.

48. Frangolias, D. D., Nakielna, E. M., Wilcox, P. G., Frangolias, D. D., Nakielna, E. M. and Wilcox, P. G. (1997) 'Pregnancy and cystic fibrosis: a case-controlled study', CHEST, 111(4), pp. 963-969.

49. Frauchiger, B. S., Binggeli, S., Yammine, S., Spycher, B., Kruger, L., Ramsey, K. A. and Latzin, P. (2021) 'Longitudinal course of clinical lung clearance index in children with cystic fibrosis', The European respiratory journal, 58(1).

50. Fuchs, H. J., Borowitz, D. S., Christiansen, D. H., Morris, E. M., Nash, M. L., Ramsey, B. W., Rosenstein, B. J., Smith, A. L. and Wohl, M. E. (1994) 'Effect of aerosolized recombinant human DNase on exacerbations of respiratory symptoms and on pulmonary function in patients with cystic fibrosis. The Pulmozyme Study Group', The New England journal of medicine, 331(10), pp. 637-42.

51. Gambazza, S., Mariani, A., Brivio, A., Carta, F., Blardone, C., Lisiero, S., Russo, M. and Colombo, C. (2022) 'Time Free From Hospitalization in Children and Adolescents With Cystic Fibrosis: Findings From FEV1, Lung Clearance Index and Peak Work Rate', Frontiers in Pediatrics, 10, pp. 926248.

52. Gondor, M., Nixon, P. A., Mutich, R., Rebovich, P. and Orenstein, D. M. (1999) 'Comparison of Flutter device and chest physical therapy in the treatment of cystic fibrosis pulmonary exacerbation', Pediatric pulmonology, 28(4), pp. 255-60.

53. Goss, C. H., Dellon, E. P., Lymp, J. F., Gibson, R., McNamara, S., Thompson, V. V., Rebovich, P., Ramsey, B. w. and Orenstein, D. M. (2011) 'Advancing patient reported outcomes in children with cystic fibrosis', Pediatric pulmonology, 46(SUPPL. 34), pp. 294-295.

54. Graham, D., O'Donnell, H. K., Sellers, D. E., Sawicki, G. S., Robinson, W. M. and Dill, E. J. (2015) 'Time spent vs episodes completed: Differences in self-reported treatment activity in surveys of CF adults', Pediatric pulmonology, 50(SUPPL. 41), pp. 433.

55. Grosse-Onnebrink, J., Mellies, U., Olivier, M., Werner, C. and Stehling, F. (2017) 'Chest physiotherapy can affect the lung clearance index in cystic fibrosis patients', Pediatric pulmonology, 52(5), pp. 625-631.

56. Grossmann, R. E., Zughaier, S. M., Kumari, M., Seydafkan, S., Lyles, R. H., Liu, S., Sueblinvong, V., Schechter, M. S., Stecenko, A. A., Ziegler, T. R. and Tangpricha, V. (2012) 'Pilot study of vitamin D supplementation in adults with cystic fibrosis pulmonary exacerbation: A randomized, controlled trial', Dermato-Endocrinology, 4(2), pp. 191-197.

57. Guigui, S., Wang, J. and Cohen, R. I. (2016) 'The use of ivacaftor in CFTR mutations resulting in residual functioning protein', Respiratory Medicine Case Reports, 19, pp. 193-195.

58. Hanssens, L., Thiebaut, I., Lefevre, N., Malfroot, A., Knoop, C., Duchateau, J. and Casimir, G. (2016) 'The clinical benefits of long-term supplementation with omega-3 fatty acids in cystic fibrosis patients - A pilot study', Prostaglandins, leukotrienes, and essential fatty acids, 108, pp. 45-50.

59. Hatziagorou, E., Avramidou, V., Kirvassilis, F. and Tsanakas, J. (2015) 'Use of lung clearance index to assess the response to intravenous treatment in cystic fibrosis', Hippokratia, 19(1), pp. 47-52.

60. Hayes, M., Yaster, M., Haythornthwaite, J. A., Riekert, K. A., Nelson McMillan, K., White, E., Mogayzel, P. J., Jr., Lechtzin, N., Hayes, M., Yaster, M., Haythornthwaite, J. A., Riekert, K. A., Nelson McMillan, K., White, E., Mogayzel, P. J., Jr. and Lechtzin, N. (2011) 'Pain is a common problem affecting clinical outcomes in adults with cystic fibrosis', CHEST, 140(6), pp. 1598-1603.

61. Henderson, D., Moore, V., MacMorran, K., Castellini, J., Hay, K., Keegan, V., Reid, D., Curtin, D. and Tay, G. (2022) 'Cohort study of sleep quality in adult patients with acute pulmonary exacerbations of cystic fibrosis', Internal Medicine Journal, 52(1), pp. 63-68.

62. Hind, D., Drabble, S. J., Arden, M. A., Mandefield, L., Waterhouse, S., Maguire, C., Cantrill, H., Robinson, L., Beever, D., Scott, A. J., Keating, S., Hutchings, M., Bradley, J., Nightingale, J., Allenby, M. I., Dewar, J., Whelan, P., Ainsworth, J., Walters, S. J. and O'Cathain, A. (2019) 'Supporting medication adherence for adults with cystic fibrosis: a randomised feasibility study', BMC Pulmonary Medicine, 19(1), pp. N.PAG-N.PAG.

63. Ho, L. P., Innes, J. A. and Greening, A. P. (1998) 'Exhaled nitric oxide is not elevated in the inflammatory airways diseases of cystic fibrosis and bronchiectasis', The European respiratory journal, 12(6), pp. 1290-4.

64. Holland, A. E., Denehy, L., Ntoumenopoulos, G., Naughton, M. T. and Wilson, J. W. (2003) 'Non-invasive ventilation assists chest physiotherapy in adults with acute exacerbations of cystic fibrosis', Thorax, 58(10), pp. 880-4.

65. Hoo, Z. H., Bramley, N. R., Curley, R., Edenborough, F. P., Walters, S. J., Campbell, M. J. and Wildman, M. J. (2019) 'Intravenous antibiotic use and exacerbation events in an adult cystic fibrosis centre: A prospective observational study', Respiratory medicine, 154, pp. 109-115.

66. Hoppe, J. E., Wagner, B. D., Sagel, S. D., Accurso, F. J. and Zemanick, E. T. (2017) 'Pulmonary exacerbations and clinical outcomes in a longitudinal cohort of infants and preschool children with cystic fibrosis', BMC Pulmonary Medicine, 17, pp. 1-8.

67. Huang, L., Lai, H. J., Antos, N., Rock, M. J., Asfour, F., Howenstine, M., Gaffin, J. M. and Farrell, P. M. (2022) 'Defining and identifying early-onset lung disease in cystic fibrosis with cumulative clinical characteristics', Pediatric pulmonology, 57(10), pp. 2363-2373.

68. Hubert, D., Marguet, C., Benichou, J., DeSouza, C., Payen-Champenois, C., Kinnman, N., Chandarana, K., Munck, A., Fajac, I., Ramel, S., Vigneron, P., Storni, V., Remus, N., Durieu, L. B. I., Laurans, M., Troussier, C. R. F., Huet, M. L. D. F., Pin, I., Payet, B. C. A., Languepin, J., Danner-Boucher, I., Mankikian, M. A. J., Wizla, N., Cosson, L., Mittaine, E. D. M., Reix, P., Dufeu, N., David, V., Bui, T. L. S., Chiron, R., Grenet, D., Chedevergne, F., Belleguic, C. and Mely, L. (2021) 'Real-World Long-Term Ivacaftor for Cystic Fibrosis in France: Clinical Effectiveness and Healthcare Resource Utilization', Pulmonary Therapy, 7(2), pp. 455-468.

69. Jafari, S.-A., Mehdizadeh-Hakkak, A., Kianifar, H.-R., Hebrani, P., Ahanchian, H. and Abbasnejad, E. (2013) 'Effects of Probiotics on Quality of Life in Children with Cystic Fibrosis; A Randomized Controlled Trial', Iranian Journal of Pediatrics, 23(6), pp. 669-674.

70. Jarad, N. A. and Sequeiros, I. M. (2012) 'A novel respiratory symptom scoring system for CF pulmonary exacerbations', QJM : monthly journal of the Association of Physicians, 105(2), pp. 137-43.

71. Jensen, J. L., Jones, C. R., Kartsonaki, C., Packer, K. A., Adler, F. R. and Liou, T. G. (2017) 'Sleep Phase Delay in Cystic Fibrosis: A Potential New Manifestation of Cystic Fibrosis Transmembrane Regulator Dysfunction', CHEST, 152(2), pp. 386-393.

72. Johnson, B., Bhanegaonkar, A., Hassan, M., Bonafede, M., Limone, B., Unni, S. and Brixner, D. (2017) 'Productivity loss associated with pulmonary exacerbations among patients with cystic fibrosis and caregivers', Journal of Managed Care and Specialty Pharmacy, 23(3-A SUPPL.), pp. S50.

73. Kanga, J., Kuhn, R., Craigmyle, L., Haverstock, D. and Church, D. (1999) 'Cystic fibrosis clinical score: a new scoring system to evaluate acute pulmonary exacerbation', Clinical therapeutics, 21(8), pp. 1343-56.

74. Kartal Ozturk, G., Eski, A., Celebi Celik, F., Conkar, S., Gulen, F., Demir, E. and Keskinoglu, A. (2020) 'Prospective evaluation of vascular changes in acute respiratory infections in children with cystic fibrosis', Turkish journal of medical sciences, 50(4), pp. 1007-1014.

75. Kazmerski, T. and Orenstein, D. M. (2012) 'The ease of breathing test tracks clinical changes in cystic fibrosis', Journal of cystic fibrosis : official journal of the European Cystic Fibrosis Society, 11(5), pp. 383-6.

76. Kelemen, L., Lee, A. L., Button, B. M., Presnell, S., Wilson, J. W. and Holland, A. E. (2012) 'Pain Impacts on Quality of Life and Interferes with Treatment in Adults with Cystic Fibrosis', Physiotherapy Research International, 17(3), pp. 132-141.

77. Kerem, E., Konstan, M. W., De Boeck, K., Accurso, F. J., Sermet-Gaudelus, I., Wilschanski, M., Elborn, J. S., Melotti, P., Bronsveld, I., Fajac, I., Malfroot, A., Rosenbluth, D. B., Walker, P. A., McColley, S. A., Knoop, C., Quattrucci, S., Rietschel, E., Zeitlin, P. L., Barth, J., Elfring, G. L., Welch, E. M., Branstrom, A., Spiegel, R. J., Peltz, S. W., Ajayi, T. and Rowe, S. M. (2014) 'Ataluren for the treatment of nonsense-mutation cystic fibrosis: a randomised, double-blind, placebo-controlled phase 3 trial', The Lancet. Respiratory medicine, 2(7), pp. 539-47.

78. Khiroya, H., Pound, R., Qureshi, U., Brown, C., Barrett, J., Rashid, R., Whitehouse, J. L., Turner, A. M. and Nash, E. F. (2014) 'Physical activity in adults with cystic fibrosis receiving intravenous antibiotics in hospital and in the community', Open Respiratory Medicine Journal, 9(1), pp. 15-21.

79. Klein, M., Cohen-Cymberknoh, M., Armoni, S., Shoseyov, D., Chisin, R., Orevi, M., Freedman, N., Kerem, E., Klein, M., Cohen-Cymberknoh, M., Armoni, S., Shoseyov, D., Chisin, R., Orevi, M., Freedman, N. and Kerem, E. (2009) '18F-fluorodeoxyglucose-PET/CT imaging of lungs in patients with cystic fibrosis', CHEST, 136(5), pp. 1220-1228.

80. A randomized double blind, placebo controlled phase 2 trial of BIIL 284 BS (an LTB4 receptor antagonist) for the treatment of lung disease in children and adults with cystic fibrosis

81. Konstan, M. W., VanDevanter, D. R., Rowe, S. M., Wilschanski, M., Kerem, E., Sermet-Gaudelus, I., DiMango, E., Melotti, P., McIntosh, J. and De Boeck, K. (2020) 'Efficacy and safety of ataluren in patients with nonsense-mutation cystic fibrosis not receiving chronic inhaled aminoglycosides: The international, randomized, double-blind, placebo-controlled Ataluren Confirmatory Trial in Cystic Fibrosis (ACT CF)', Journal of cystic fibrosis : official journal of the European Cystic Fibrosis Society, 19(4), pp. 595-601.

82. Kotha, K., Szczesniak, R. D., Naren, A. P., Fenchel, M. C., Duan, L. L., McPhail, G. L. and Clancy, J. P. (2015) 'Concentration of fractional excretion of nitric oxide (FENO): A potential airway biomarker of restored CFTR function', Journal of cystic fibrosis : official journal of the European Cystic Fibrosis Society, 14(6), pp. 733-40.

83. Kruizinga, M. D., Essers, E., Stuurman, F. E., Yavuz, Y., de Kam, M. L., Zhuparris, A., Janssens, H. M., Groothuis, I., Sprij, A. J., Nuijsink, M., Cohen, A. F. and Driessen, G. J. A. (2022) 'Clinical validation of digital biomarkers for paediatric patients with asthma and cystic fibrosis: potential for clinical trials and clinical care', The European respiratory journal, 59(6).

84. Laube, B. L., Chang, D. Y., Blask, A. N. and Rosenstein, B. J. (1992) 'Radioaerosol assessment of lung improvement in cystic fibrosis patients treated for acute pulmonary exacerbations', CHEST, 101(5), pp. 1302-1308.

85. Lechtzin, N., Allgood, S., Hong, G., Riekert, K., Haythornthwaite, J. A., Mogayzel, P., Hankinson, J. and Yaster, M. (2016) 'The Association Between Pain and Clinical Outcomes in Adolescents With Cystic Fibrosis', Journal of Pain & Symptom Management, 52(5), pp. 681-687.

86. Lechtzin, N., West, N., Allgood, S., Wilhelm, E., Khan, U., Mayer-Hamblett, N., Aitken, M. L., Ramsey, B. W., Boyle, M. P., Mogayzel, P. J., Jr. and Goss, C. H. (2013) 'Rationale and design of a randomized trial of home electronic symptom and lung function monitoring to detect cystic fibrosis pulmonary exacerbations: the early intervention in cystic fibrosis exacerbation (eICE) trial', Contemporary clinical trials, 36(2), pp. 460-9.

87. Loeve, M., Gerbrands, K., Hop, W., Rosenfeld, M. and Tiddens, H. (2009) 'Bronchiectasis on chest computed tomography (CT) is a strong predictor of respiratory tract exacerbations in cystic fibrosis (CF)', Journal of Thoracic Imaging, 24(3), pp. w13.

88. Lucca, F., Da Dalt, L., Ros, M., Gucciardi, A., Pirillo, P., Naturale, M., Perilongo, G., Giordano, G. and Baraldi, E. (2018) 'Asymmetric dimethylarginine and related metabolites in exhaled breath condensate of children with cystic fibrosis', The clinical respiratory journal, 12(1), pp. 140-148.

89. Mayer, O. H., Jawad, A. F., McDonough, J. and Allen, J. (2008) 'Lung function in 3-5-year-old children with cystic fibrosis', Pediatric pulmonology, 43(12), pp. 1214-23.

90. Mayer-Hamblett, N., Nichols, D. P., Odem-Davis, K., Riekert, K. A., Sawicki, G. S., Donaldson, S. H., Ratjen, F., Konstan, M. W., Simon, N., Rosenbluth, D. B., Retsch-Bogart, G., Clancy, J. P., Van Dalfsen, J. M., Buckingham, R. and Gifford, A. H. (2021) 'Evaluating the impact of stopping chronic therapies after modulator drug therapy in cystic fibrosis: The SIMPLIFY clinical trial study design', Annals of the American Thoracic Society, 18(8), pp. 1397-1405.

91. Mayer-Hamblett, N., Retsch-Bogart, G., Kloster, M., Accurso, F., Rosenfeld, M., Albers, G., Black, P., Brown, P., Cairns, A., Davis, S. D., Graff, G. R., Kerby, G. S., Orenstein, D., Buckingham, R. and Ramsey, B. W. (2018) 'Azithromycin for Early Pseudomonas Infection in Cystic Fibrosis. The OPTIMIZE Randomized Trial', American Journal of Respiratory & Critical Care Medicine, 198(9), pp. 1177-1187.

92. Mc Closkey, M., Redmond, A. O. B., Mc Cabe, C., Pyper, S., Westerterp, K. R. and Elborn, S. J. (2004) 'Energy balance in cystic fibrosis when stable and during a respiratory exacerbation', Clinical nutrition (Edinburgh, Scotland), 23(6), pp. 1405-12.

93. McIlwaine, M. P., Alarie, N., Davidson, G. F., Lands, L. C., Ratjen, F., Milner, R., Owen, B. and Agnew, J. L. (2013) 'Long-term multicentre randomised controlled study of high frequency chest wall oscillation versus positive expiratory pressure mask in cystic fibrosis', Thorax, 68(8), pp. 746-751.

94. Middleton, A., Robinson, P., McKay, K. and Selvadurai, H. (2012) 'Pilot study of inhaled dry powder mannitol in young people with CF hospitalised with pulmonary exacerbation', Pediatric pulmonology, 47(SUPPL. 35), pp. 355-356.

95. Middleton, P. G., Mall, M. A., Drevinek, P., Lands, L. C., McKone, E. F., Polineni, D., Ramsey, B. W., Taylor-Cousar, J. L., Tullis, E., Vermeulen, F., Marigowda, G., McKee, C. M., Moskowitz, S. M., Nair, N., Savage, J., Simard, C., Tian, S., Waltz, D., Xuan, F. and Rowe, S. M. (2019) 'Elexacaftor-Tezacaftor-Ivacaftor for Cystic Fibrosis with a Single Phe508del Allele', New England Journal of Medicine, 381(19), pp. 1809-1819.

96. Mikesell, C. L., Kempainen, R. R., Laguna, T. A., Menk, J. S., Wey, A. R., Gaillard, P. R. and Regelmann, W. E. (2017) 'Objective Measurement of Adherence to Out-Patient Airway Clearance Therapy by High-Frequency Chest Wall Compression in Cystic Fibrosis', Respiratory Care, 62(7), pp. 920-927.

97. Monteiro, K. S., Azevedo, M. d. P., Jales, L. M., da Silva, F. E. P., Arrais, R. F. and de Mendonça, K. M. P. P. (2019) 'Effects of aerobic interval training on glucose tolerance in children and adolescents with cystic fibrosis: a randomized trial protocol', Trials, 20(1), pp. 1-9.

98. Mozzillo, E., Franzese, A., Valerio, G., Sepe, A., De Simone, I., Mazzarella, G., Ferri, P. and Raia, V. (2009) 'One-year glargine treatment can improve the course of lung disease in children and adolescents with cystic fibrosis and early glucose derangements', Pediatric Diabetes, 10(3), pp. 162-167.

99. Munoz, G., de Gracia, J., Giron, R., Olveira, C., Alvarez, A., Buxo, M., Birring, S. S. and Vendrell, M. (2021) 'Validation of a Spanish version of the Leicester Cough Questionnaire in cystic fibrosis', Chronic respiratory disease, 18, pp. 14799731211036903.

100. McKone, E. F., Borowitz, D., Drevinek, P., Griese, M., Konstan, M. W., Wainwright, C., Ratjen, F., Sermet-Gaudelus, I., Plant, B., Munck, A., Jiang, Y., Gilmartin, G. and Davies, J. C. (2014) 'Long-term safety and efficacy of ivacaftor in patients with cystic fibrosis who have the Gly551Asp-CFTR mutation: a phase 3, open-label extension study (PERSIST)', The Lancet. Respiratory medicine, 2(11), pp. 902-910.

101. Naon, H., Hack, S., Shelton, M. T., Gotthoffer, R. C. and Gozal, D. (1993) 'Resting energy expenditure. Evolution during antibiotic treatment for pulmonary exacerbation in cystic fibrosis', CHEST, 103(6), pp. 1819-1825.

102. Nash, E. F., Choyce, J., Carrolan, V., Justice, E., Shaw, K. L., Sitch, A., Mistry, H. and Whitehouse, J. L. (2022) 'A prospective randomised controlled mixed-methods pilot study of home monitoring in adults with cystic fibrosis', Therapeutic advances in respiratory disease, 16, pp. 17534666211070133.

103. Neemuchwala, F., Ghadimi Mahani, M., Pang, Y., Lee, E., Johnson, T. D., Galbán, C. J., Fortuna, A. B., Sanchez-Jacob, R., Flask, C. A. and Nasr, S. Z. (2020) 'Lung T1 mapping magnetic resonance imaging in the assessment of pulmonary disease in children with cystic fibrosis: a pilot study', Pediatric Radiology, 50(7), pp. 923-934.

104. Newport, S., Amin, N. and Dozor, A. J. (2009) 'Exhaled breath condensate pH and ammonia in cystic fibrosis and response to treatment of acute pulmonary exacerbations', Pediatric pulmonology, 44(9), pp. 866-72.

105. Nicholson, T. T., Barry, P. J., Waterhouse, D. F., Nolan, G. M., McKone, E. F. and Gallagher, C. G. (2017) 'Relationship between pulmonary hyperinflation and dyspnoea severity during acute exacerbations of cystic fibrosis', Respirology (Carlton, Vic.), 22(1), pp. 141-148.

106. Ojoo, J. C., Mulrennan, S. A., Kastelik, J. A., Morice, A. H. and Redington, A. E. (2005) 'Exhaled breath condensate pH and exhaled nitric oxide in allergic asthma and in cystic fibrosis', Thorax, 60(1), pp. 22-6.

107. Paff, T., van der Schee, M. P., Daniels, J. M. A., Pals, G., Postmus, P. E., Sterk, P. J. and Haarman, E. G. (2013) 'Exhaled molecular profiles in the assessment of cystic fibrosis and primary ciliary dyskinesia', Journal of cystic fibrosis : official journal of the European Cystic Fibrosis Society, 12(5), pp. 454-60.

108. Tate, S., MacGregor, G., Davis, M., Innes, J. A. and Greening, A. P. (2002) 'Airways in cystic fibrosis are acidified: detection by exhaled breath condensate', Thorax, 57(11), pp. 926-9.

109. van Horck, M., Smolinska, A., Wesseling, G., de Winter-de Groot, K., de Vreede, I., Winkens, B., Jobsis, Q., Dallinga, J., Dompeling, E. and van Schooten, F.-J. (2021) 'Exhaled volatile organic compounds detect pulmonary exacerbations early in children with cystic fibrosis: results of a 1 year observational pilot study', Journal of breath research, 15(2), pp. 026012.

110. Pallin, M., Kumar, S., Daley, C., Dawadi, S., Leong, P., Carr, E. and Soldatos, G. (2021) 'Continuous glucose monitoring indices predict poor FEV1 recovery following cystic fibrosis pulmonary exacerbations', Journal of cystic fibrosis : official journal of the European Cystic Fibrosis Society, 20(5), pp. 785-791.

111. Phillips, G. E., Pike, S. E., Jaffe, A. and Bush, A. (2004) 'Comparison of active cycle of breathing and high-frequency oscillation jacket in children with cystic fibrosis', Pediatric pulmonology, 37(1), pp. 71-5.

112. Quan, J. M., Tiddens, H. A., Sy, J. P., McKenzie, S. G., Montgomery, M. D., Robinson, P. J., Wohl, M. E. and Konstan, M. W. (2001) 'A two-year randomized, placebo-controlled trial of dornase alfa in young patients with cystic fibrosis with mild lung function abnormalities', The Journal of pediatrics, 139(6), pp. 813-20.

113. Quittner, A. L., Modi, A. C., Wainwright, C., Otto, K., Kirihara, J., Montgomery, A. B., Quittner, A. L., Modi, A. C., Wainwright, C., Otto, K., Kirihara, J. and Montgomery, A. B. (2009) 'Determination of the minimal clinically important difference scores for the Cystic Fibrosis Questionnaire-Revised respiratory symptom scale in two populations of patients with cystic fibrosis and chronic Pseudomonas aeruginosa airway infection', CHEST, 135(6), pp. 1610-1618.

114. Ramsey, B. W., Davies, J., McElvaney, N. G., Tullis, E., Bell, S. C., Devínek, P., Griese, M., McKone, E. F., Wainwright, C. E., Konstan, M. W., Moss, R., Ratjen, F., Sermet-Gaudelus, I., Rowe, S. M., Dong, Q., Rodriguez, S., Yen, K., Ordoñez, C., Elborn, J. S. and Ramsey, B. W. (2011) 'A CFTR potentiator in patients with cystic fibrosis and the G551D mutation', New England Journal of Medicine, 365(18), pp. 1663-1672.

115. Rayment, J. H., Couch, M. J., McDonald, N., Kanhere, N., Manson, D., Santyr, G. and Ratjen, F. (2019) 'Hyperpolarised 129Xe magnetic resonance imaging to monitor treatment response in children with cystic fibrosis', The European respiratory journal, 53(5).

116. Rayment, J. H., Stanojevic, S., Davis, S. D., Retsch-Bogart, G. and Ratjen, F. (2018) 'Lung clearance index to monitor treatment response in pulmonary exacerbations in preschool children with cystic fibrosis', Thorax, 73(5), pp. 451-458.

117. Reilly, J. J., Ralston, J. M., Paton, J. Y., Edwards, C. A., Weaver, L. T., Wilkinson, J. and Evans, T. J. (1999) 'Energy balance during acute respiratory exacerbations in children with cystic fibrosis', The European respiratory journal, 13(4), pp. 804-9.

118. Renz, D. M., Scholz, O., Bottcher, J., Maurer, M. H., Denecke, T., Schwarz, C., Pfeil, A., Streitparth, F., Huppertz, A., Mehl, A., Poellinger, A., Staab, D., Hamm, B. and Mentzel, H.-J. (2015) 'Comparison between magnetic resonance imaging and computed tomography of the lung in patients with cystic fibrosis with regard to clinical, laboratory, and pulmonary functional parameters', Investigative radiology, 50(10), pp. 733-42.

119. Robinson, P., Schechter, M. S., Sly, P. D., Winfield, K., Smith, J., Brennan, S., Shinkai, M., Henke, M. O. and Rubin, B. K. (2012) 'Clarithromycin therapy for patients with cystic fibrosis: a randomized controlled trial', Pediatric pulmonology, 47(6), pp. 551-7.

120. Robinson, P. D., Cooper, P., Van Asperen, P., Fitzgerald, D. and Selvadurai, H. (2009) 'Using index of ventilation to assess response to treatment for acute pulmonary exacerbation in children with cystic fibrosis', Pediatric pulmonology, 44(8), pp. 733-42.

121. Robinson, P. J. (2002) 'Dornase alfa in early cystic fibrosis lung disease', Pediatric pulmonology, 34(3), pp. 237-41.

122. Rosenfeld, M., Emerson, J., Williams-Warren, J., Pepe, M., Smith, A., Montgomery, A. B. and Ramsey, B. (2001) 'Defining a pulmonary exacerbation in cystic fibrosis', Journal of Pediatrics, 139(3), pp. 359-365.

123. Rosenfeld, M., Ratjen, F., Brumback, L., Daniel, S., Rowbotham, R., McNamara, S., Johnson, R., Kronmal, R., Davis, S. D., Rosenfeld, M., Ratjen, F., Brumback, L., Daniel, S., Rowbotham, R., McNamara, S., Johnson, R., Kronmal, R. and Davis, S. D. (2012) 'Inhaled hypertonic saline in infants and children younger than 6 years with cystic fibrosis: the ISIS randomized controlled trial', JAMA: Journal of the American Medical Association, 307(21), pp. 2269-2277.

124. Sagel, S. D., Khan, U., Jain, R., Graff, G., Daines, C. L., Dunitz, J. M., Borowitz, D., Orenstein, D. M., Abdulhamid, I., Noe, J. and et al. (2018) 'Effects of an Antioxidant-enriched Multivitamin in Cystic Fibrosis. A Randomized, Controlled, Multicenter Clinical Trial', American journal of respiratory and critical care medicine, 198(5), pp. 639-647.

125. Saiman, L., Anstead, M., Mayer-Hamblett, N., Lands, L. C., Kloster, M., Hocevar-Trnka, J., Goss, C. H., Rose, L. M., Burns, J. L., Marshall, B. C., Ratjen, F., Saiman, L., Anstead, M., Mayer-Hamblett, N., Lands, L. C., Kloster, M., Hocevar-Trnka, J., Goss, C. H., Rose, L. M. and Burns, J. L. (2010) 'Effect of azithromycin on pulmonary function in patients with cystic fibrosis uninfected with Pseudomonas aeruginosa: a randomized controlled trial', JAMA: Journal of the American Medical Association, 303(17), pp. 1707-1715.

126. Saiman, L., Marshall, B. C., Mayer-Hamblett, N., Burns, J. L., Quittner, A. L., Cibene, D. A., Coquillette, S., Fieberg, A. Y., Accurso, F. J., Campbell, P. W., III, Saiman, L., Marshall, B. C., Mayer-Hamblett, N., Burns, J. L., Quittner, A. L., Cibene, D. A., Coquillette, S., Fieberg, A. Y., Accurso, F. J. and Campbell, P. W., 3rd (2003) 'Azithromycin in patients with cystic fibrosis chronically infected with Pseudomonas aeruginosa: a randomized controlled trial', JAMA: Journal of the American Medical Association, 290(13), pp. 1749-1756.

127. Sakarya, A., Uyan, Z. S., Baydemir, C., Anik, Y., Erdem, E., Gokdemir, Y., Karadag, B., Karakoc, F. and Ersu, R. (2016) 'Evaluation of children with cystic fibrosis by impulse oscillometry when stable and at exacerbation', Pediatric pulmonology, 51(11), pp. 1151-1158.

128. Sanders, D. B., Goss, C. H., Van Devanter, D. R., Heltse, S. L., Thompson, V. and Flume, P. (2015) 'Standardized treatment of pulmonary exacerbations (STOP) study: Clinical presentations of pulmonary exacerbations', Journal of Cystic Fibrosis, 14(SUPPL. 1), pp. S106.

129. Sanders, D. B., Solomon, G. M., Beckett, V. V., West, N. E., Daines, C. L., Heltshe, S. L., VanDevanter, D. R., Spahr, J. E., Gibson, R. L., Nick, J. A., Marshall, B. C., Flume, P. A. and Goss, C. H. (2017) 'Standardized Treatment of Pulmonary Exacerbations (STOP) study: Observations at the initiation of intravenous antibiotics for cystic fibrosis pulmonary exacerbations', Journal of cystic fibrosis : official journal of the European Cystic Fibrosis Society, 16(5), pp. 592-599.

130. Sawicki, G. S., Chilvers, M., McNamara, J., Naehrlich, L., Saunders, C., Sermet-Gaudelus, I., Wainwright, C. E., Ahluwalia, N., Campbell, D., Harris, R. S., Paz-Diaz, H., Shih, J. L. and Davies, J. C. (2022) 'A Phase 3, open-label, 96-week trial to study the safety, tolerability, and efficacy of tezacaftor/ivacaftor in children ≥ 6 years of age homozygous for F508del or heterozygous for F508del and a residual function CFTR variant', Journal of Cystic Fibrosis, 21(4), pp. 675-683.

131. Sc, N. N. M., Shoseyov, D., Kerem, E. and Zangen, D. H. (2010) 'Patients with cystic fibrosis and normoglycemia exhibit diabetic glucose tolerance during pulmonary exacerbation', Journal of cystic fibrosis : official journal of the European Cystic Fibrosis Society, 9(3), pp. 199-204.

132. Schmid-Mohler, G., Caress, A.-L., Spirig, R., Benden, C. and Yorke, J. (2019) '"Thrust out of normality"-How adults living with cystic fibrosis experience pulmonary exacerbations: A qualitative study', Journal of clinical nursing, 28(1-2), pp. 190-200.

133. Scholz, O., Denecke, T., Böttcher, J., Schwarz, C., Mentzel, H. J., Streitparth, F., Maurer, M. H., Pfeil, A., Huppertz, A., Mehl, A., Staab, D., Hamm, B. and Renz, D. M. (2017) 'MRI of cystic fibrosis lung manifestations: sequence evaluation and clinical outcome analysis', Clinical Radiology, 72(9), pp. 754-763.

134. Schwarz, C., Grehn, C., Temming, S., Holz, F. and Eschenhagen, P.N., 2021. Clinical impact of levofloxacin inhalation solution in cystic fibrosis patients in a real-world setting. Journal of Cystic Fibrosis, 20(6), pp.1035-1039.

135. Sequeiros, I. M. and Jarad, N. (2012) 'Factors associated with a shorter time until the next pulmonary exacerbation in adult patients with cystic fibrosis', Chronic respiratory disease, 9(1), pp. 9-16.

136. Sequeiros, I. M. and Jarad, N. A. (2012) 'Extending the course of intravenous antibiotics in adult patients with cystic fibrosis with acute pulmonary exacerbations', Chronic respiratory disease, 9(4), pp. 213-20.

137. Sackey, A. H., Taylor, C. J., Barraclough, M., Wales, J. K. H. and Pickering, M. (1995) 'Growth hormone as a nutritional adjunct in cystic fibrosis: Results of a pilot study', Journal of Human Nutrition and Dietetics, 8(3), pp. 185-191.

138. Shah, R. M., Sexauer, W., Ostrum, B. J., Fiel, S. B. and Friedman, A. C. (1997) 'High-resolution CT in the acute exacerbation of cystic fibrosis: evaluation of acute findings, reversibility of those findings, and clinical correlation', AJR. American journal of roentgenology, 169(2), pp. 375-80.

139. Short, C., Semple, T., Saunders, C., Hughes, D., Irving, S., Gardener, L., Rosenthal, M., Robinson, P. D. and Davies, J. C. (2022) 'A Short extension to multiple breath washout provides additional signal of distal airway disease in people with CF: A pilot study', Journal of cystic fibrosis : official journal of the European Cystic Fibrosis Society, 21(1), pp. 146-154.

140. FitzMaurice, T. S., McCann, C., Nazareth, D. S., McNamara, P. S. and Walshaw, M. J. (2022) 'Use of Dynamic Chest Radiography to Assess Treatment of Pulmonary Exacerbations in Cystic Fibrosis', Radiology, 303(3), pp. 675-681.

141. Singh, S., Hornick, D., Fedler, J., Launspach, J. L., Teresi, M. E., Santacroce, T. R., Cavanaugh, J. E., Horan, R., Nelson, G., Starner, T. D., Zabner, J. and Durairaj, L. (2020) 'Randomized controlled study of aerosolized hypertonic xylitol versus hypertonic saline in hospitalized patients with pulmonary exacerbation of cystic fibrosis', Journal of cystic fibrosis : official journal of the European Cystic Fibrosis Society, 19(1), pp. 108-113.

142. Smith, J. A., Owen, E. C., Jones, A. M., Dodd, M. E., Webb, A. K. and Woodcock, A. (2006) 'Objective measurement of cough during pulmonary exacerbations in adults with cystic fibrosis', Thorax, 61(5), pp. 425-9.

143. Smyth, A., Tan, K. H. V., Hyman-Taylor, P., Mulheran, M., Lewis, S., Stableforth, D. and Knox, A. (2005) 'Once versus three-times daily regimens of tobramycin treatment for pulmonary exacerbations of cystic fibrosis - The TOPIC study: A randomised controlled trial', Lancet, 365(9459), pp. 573-578.

144. Somerville, L., List, R., Compton, M., Bruschwein, H., Jennings, D., Murray, R., Starheim, E., Webb, K., Kalmanek, J., Gettle, L. and Albon, D. (2021) '110 Real-world outcomes in cystic fibrosis telehealth clinical care during the COVID-19 pandemic', Journal of Cystic Fibrosis, 20(Supplement 2), pp. S55-S56.

145. Sonneveld, N., Stanojevic, S., Jensen, R., Subbarao, P. and Ratjen, F. (2015) 'Does the lung clearance index track with disease progression in early childhood?', European Respiratory Journal, 46(SUPPL. 59).

146. Stahl, M., Steinke, E., Graeber, S. Y., Joachim, C., Seitz, C., Kauczor, H.-U., Eichinger, M., Hämmerling, S., Sommerburg, O., Wielpüz, M. O., Mall, M. A. and Wielpütz, M. O. (2021) 'Magnetic Resonance Imaging Detects Progression of Lung Disease and Impact of Newborn Screening in Preschool Children with Cystic Fibrosis', American Journal of Respiratory & Critical Care Medicine, 204(8), pp. 943-953.

147. Stahl M, Wielpütz MO, Ricklefs I, et al. Preventive Inhalation of Hypertonic Saline in Infants with Cystic Fibrosis (PRESIS). A Randomized, Double-Blind, Controlled Study. Am J Respir Crit Care Med. 2019;199(10):1238-1248. doi:10.1164/rccm.201807-1203OC

148. Stallings, V. A., Fung, E. B., Hofley, P. M. and Scanlin, T. F. (1998) 'Acute pulmonary exacerbation is not associated with increased energy expenditure in children with cystic fibrosis', The Journal of pediatrics, 132(3 Pt 1), pp. 493-9.

149. Stuart Elborn, J., Geller, D. E., Conrad, D., Aaron, S. D., Smyth, A. R., Fischer, R., Kerem, E., Bell, S. C., Loutit, J. S., Dudley, M. N., Morgan, E. E., VanDevanter, D. R. and Flume, P. A. (2015) 'A phase 3, open-label, randomized trial to evaluate the safety and efficacy of levofloxacin inhalation solution (APT-1026) versus tobramycin inhalation solution in stable cystic fibrosis patients', Journal of cystic fibrosis : official journal of the European Cystic Fibrosis Society, 14(4), pp. 507-14.

150. Suri, R., Metcalfe, C., Lees, B., Grieve, R., Flather, M., Normand, C., Thompson, S., Bush, A. and Wallis, C. (2001) 'Comparison of hypertonic saline and alternate-day or daily recombinant human deoxyribonuclease in children with cystic fibrosis: a randomised trial', Lancet (London, England), 358(9290), pp. 1316-21.

151. Suri, R., Metcalfe, C., Wallis, C. and Bush, A. (2007) 'Assessing the usefulness of outcomes measured in a cystic fibrosis treatment trial', Respiratory medicine, 101(2), pp. 254-60.

152. Tangpricha, V., Lukemire, J., Chen, Y., Binongo, J. N. G., Judd, S. E., Michalski, E. S., Lee, M. J., Walker, S., Ziegler, T. R., Tirouvanziam, R., Zughaier, S. M., Chesdachai, S., Hermes, W. A., Chmiel, J. F., Grossmann, R. E., Gaggar, A., Joseph, P. M. and Alvarez, J. A. (2019) 'Vitamin D for the Immune System in Cystic Fibrosis (DISC): a double-blind, multicenter, randomized, placebo-controlled clinical trial', American Journal of Clinical Nutrition, 109(3), pp. 544-553.

153. Tepper, L. A., Ciet, P., Caudri, D., Quittner, A. L., Utens, E. M. W. J. and Tiddens, H. A. W. M. (2016) 'Validating chest MRI to detect and monitor cystic fibrosis lung disease in a pediatric cohort', Pediatric pulmonology, 51(1), pp. 34-41.

154. Tepper, L. A., Utens, E. M. W. J., Caudri, D., Bos, A. C., Gonzalez-Graniel, K., Duivenvoorden, H. J., van der Wiel, E. C. W., Quittner, A. L. and Tiddens, H. A. W. M. (2013) 'Impact of bronchiectasis and trapped air on quality of life and exacerbations in cystic fibrosis', The European respiratory journal, 42(2), pp. 371-9.

155. Thee, S., Stahl, M., Fischer, R., Sutharsan, S., Ballmann, M., Müller, A., Lorenz, D., Urbanski-Rini, D., Püschner, F., Amelung, V. E., Fuchs, C. and Mall, M. A. (2021) 'A multi-centre, randomized, controlled trial on coaching and telemonitoring in patients with cystic fibrosis: conneCT CF', BMC Pulmonary Medicine, 21(1), pp. 1-11.

156. Treggiari, M. M., Retsch-Bogart, G., Mayer-Hamblett, N., Khan, U., Kulich, M., Kronmal, R., Williams, J., Hiatt, P., Gibson, R. L., Spencer, T., Orenstein, D., Chatfield, B. A., Froh, D. K., Burns, J. L., Rosenfeld, M. and Ramsey, B. W. (2011) 'Comparative efficacy and safety of 4 randomized regimens to treat early Pseudomonas aeruginosa infection in children with cystic fibrosis', Archives of pediatrics & adolescent medicine, 165(9), pp. 847-56.

157. Treggiari, M. M., Rosenfeld, M., Mayer-Hamblett, N., Retsch-Bogart, G., Gibson, R. L., Williams, J., Emerson, J., Kronmal, R. A. and Ramsey, B. W. (2009) 'Early anti-pseudomonal acquisition in young patients with cystic fibrosis: rationale and design of the EPIC clinical trial and observational study'', Contemporary clinical trials, 30(3), pp. 256-68.

158. Van Biervliet, S., Hauser, B., Verhulst, S., Stepman, H., Delanghe, J., Warzee, J.-P., Pot, B., Vandewiele, T. and Wilschanski, M. (2018) 'Probiotics in cystic fibrosis patients: A double blind crossover placebo controlled study: Pilot study from the ESPGHAN Working Group on Pancreas/CF', Clinical nutrition ESPEN, 27, pp. 59-65.

159. Horck, M., Winkens, B., Wesseling, G., Winter-de Groot, K., Vreede, I., Jöbsis, Q., Dompeling, E., van Horck, M., de Winter-de Groot, K., de Vreede, I. and Jöbsis, Q. (2017) 'Factors associated with changes in health-related quality of life in children with cystic fibrosis during 1-year follow-up', European Journal of Pediatrics, 176(8), pp. 1047-1054.

160. van Horck, M., Winkens, B., Wesseling, G., van Vliet, D., van de Kant, K., Vaassen, S., de Winter-de Groot, K., de Vreede, I., Jobsis, Q. and Dompeling, E. (2017) 'Early detection of pulmonary exacerbations in children with Cystic Fibrosis by electronic home monitoring of symptoms and lung function', Scientific reports, 7(1), pp. 12350.

161. Vermeulen, F., Proesmans, M., Boon, M., Havermans, T. and De Boeck, K. (2014) 'Lung clearance index predicts pulmonary exacerbations in young patients with cystic fibrosis', Thorax, 69(1), pp. 39-45.

162. Voldby, C., Green, K., Philipsen, L., Sandvik, R.M., Skov, M., Buchvald, F., Pressler, T. and Nielsen, K.G., 2021. Withdrawal of dornase alfa increases ventilation inhomogeneity in children with cystic fibrosis. Journal of Cystic Fibrosis, 20(6), pp.949-956.

163. Walicka-Serzysko, K., Postek, M., Jeneralska, N., Cichocka, A., Milczewska, J. and Sands, D., 2021. The effects of the addition of a new airway clearance device to chest physiotherapy in children with cystic fibrosis pulmonary exacerbations. Journal of Mother and Child, 24(3), pp.16-24.

164. Wang J. (2017) ‘Efficacy of lumacaftor/ivacaftor in patients with mild lung disease’, Pediatric pulmonology., 52(Supplement 47), .

165. Ward, N., Stiller, K., Rowe, H., Morrow, S., Morton, J., Greville, H. and Holland, A.E., 2018. Airway clearance by exercising in mild cystic fibrosis (ACE-CF): a feasibility study. Respiratory medicine, 142, pp.23-28.

166. Waterhouse, D. F., mclaughlin, A. M. And Gallagher, C. G. (2009) 'Time course and recovery of arterial blood gases during exacerbations in adults with Cystic Fibrosis', Journal of Cystic Fibrosis, 8(1), pp. 9-13.

167. Welsh, L., Nesci, C., Tran, H., Tomai, M. And Ranganathan, S. (2014) 'Lung clearance index during hospital admission in school-age children with cystic fibrosis', Journal of Cystic Fibrosis, 13(6), pp. 687-691.

168. Widger, J., Oliver, M.R., O’Connell, M., Cameron, F.J., Ranganathan, S. and Robinson, P.J., 2012. Glucose tolerance during pulmonary exacerbations in children with cystic fibrosis.

169. Williams, M.T., Parsons, D.W., Frick, R.A., Ellis, E.R., Martin, A.J., Giles, S.E. and Grant, E.R., 2001. Acute respiratory infection in patients with cystic fibrosis with mild pulmonary impairment: comparison of two physiotherapy regimens. Australian journal of physiotherapy, 47(4), pp.227-236.

170. Wilmott, R.W., Amin, R.S., Colin, A.A., devault, A., Dozor, A.J., Eigen, H., Johnson, C., Lester, L.A., mccoy, K., mckean, L.P. and Moss, R., 1996. Aerosolized recombinant human dnase in hospitalized cystic fibrosis patients with acute pulmonary exacerbations. American journal of respiratory and critical care medicine, 153(6), pp.1914-1917.

171. Wolter, J., Seeney, S., Bell, S., Bowler, S., Masel, P. and McCormack, J., 2002. Effect of long term treatment with azithromycin on disease parameters in cystic fibrosis: a randomised trial. Thorax, 57(3), pp.212-216.

172. Wood, J., Jenkins, S., Putrino, D., Mulrennan, S., Morey, S., Cecins, N., Bear, N. and Hill, K., 2020. A smartphone application for reporting symptoms in adults with cystic fibrosis improves the detection of exacerbations: Results of a randomised controlled trial. Journal of Cystic Fibrosis, 19(2), pp.271-276.

173. Yi, M. S., Tsevat, J., Wilmott, R. W., Kotagal, U. R. And Britto, M. T. (2004) 'The impact of treatment of pulmonary exacerbations on the health-related quality of life of patients with cystic fibrosis: does hospitalization make a difference?', Journal of Pediatrics, 144(6), pp. 711-718.

174. Zemke, A.C., Nouraie, S.M., Moore, J., Gaston, J.R., Rowan, N.R., Pilewski, J.M., Bomberger, J.M. and Lee, S.E., 2019, July. Clinical predictors of cystic fibrosis chronic rhinosinusitis severity. In International forum of allergy & rhinology (Vol. 9, No. 7, pp. 759-765).

175. Adeboyeku, D., Jones, A.L. and Hodson, M.E., 2011. Twice vs three-times daily antibiotics in the treatment of pulmonary exacerbations of cystic fibrosis. Journal of Cystic Fibrosis, 10(1), pp.25-30.

176. Antuni, J.D., Kharitonov, S.A., Hughes, D., Hodson, M.E. and Barnes, P.J., 2000. Increase in exhaled carbon monoxide during exacerbations of cystic fibrosis. Thorax, 55(2), pp.138-142.

177. Equi, A., Balfour-Lynn, I.M., Bush, A. and Rosenthal, M., 2002. Long term azithromycin in children with cystic fibrosis: a randomised, placebo-controlled crossover trial. The Lancet, 360(9338), pp.978-984.

178. Flume PA, Biner RF, Downey DG, et al. Long-term safety and efficacy of tezacaftor-ivacaftor in individuals with cystic fibrosis aged 12 years or older who are homozygous or heterozygous for Phe508del CFTR (EXTEND): an open-label extension study [published correction appears in Lancet Respir Med. 2021 Apr;9(4):e38]. Lancet Respir Med. 2021;9(7):733-746. doi:10.1016/S2213-2600(20)30510-5

179. Ratjen, F., Hug, C., Marigowda, G., Tian, S., Huang, X., Stanojevic, S., Milla, C. E., Robinson, P. D., Waltz, D., Davies, J. C., Rosenfeld, M., Starner, T., Retsch-Bogart, G., Chmiel, J., Orenstein, D., Milla, C., Rubenstein, R., Walker, S., Cornell, A., Asfour, F., Black, P., Colombo, J., Froh, D., McColley, S., Ruiz, F., Quintero, D., Casey, A., Mueller, G., Flume, P., Livingston, F., Rock, M., O'Sullivan, B., Schmidt, H., Lahiri, T., McNamara, J., Chidekel, A., Sass, L., Keens, T., Schaeffer, D., Solomon, M., Chilvers, M., Lands, L., Junge, S., Griese, M., Staab, D., Pressler, T., van Koningsburggen-Rietschel, S., Naehrlich, L., Reid, A., Balfour-Lynn, I., Urquhart, D., Lee, T., Munck, A., Gaudelus, I. S., De Boeck, C., Reix, P., Malfroot, A., Bui, S., Selvadurai, H., Robinson, P., Wainwright, C., Clements, B., Hilton, J. and Hjelte, L. (2017) 'Efficacy and safety of lumacaftor and ivacaftor in patients aged 6–11 years with cystic fibrosis homozygous for F508del-CFTR: a randomised, placebo-controlled phase 3 trial', The Lancet Respiratory Medicine, 5(7), pp. 557-567.

180. Chilvers, M. A., Davies, J. C., Milla, C., Tian, S., Han, Z., Cornell, A. G., Owen, C. A. and Ratjen, F. (2021) 'Long-term safety and efficacy of lumacaftor-ivacaftor therapy in children aged 6-11 years with cystic fibrosis homozygous for the F508del-CFTR mutation: a phase 3, open-label, extension study', The Lancet. Respiratory medicine, 9(7), pp. 721-732.
181. Taylor-Cousar, J. L., Munck, A., McKone, E. F., van der Ent, C. K., Moeller, A., Simard, C., Wang, L. T., Ingenito, E. P., McKee, C., Lu, Y., Lekstrom-Himes, J. and Elborn, J. S. (2017) 'Tezacaftor-Ivacaftor in Patients with Cystic Fibrosis Homozygous for Phe508del', The New England journal of medicine, 377(21), pp. 2013-2023.
182. Gozal, D., Bailey, S. L. and Keens, T. G. (1993) 'Evolution of pulmonary function during an acute exacerbation in hospitalized patients with cystic fibrosis', Pediatric Pulmonology, 16(6), pp. 347-53.
183. Bilton, D., Fajac, I., Pressler, T., Clancy, J. P., Sands, D., Minic, P., Cipolli, M., Galeva, I., Sole, A., Quittner, A. L., Jumadilova, Z., Ciesielska, M. and Konstan, M. W. (2021) 'Long-term amikacin liposome inhalation suspension in cystic fibrosis patients with chronic P. aeruginosa infection', Journal of Cystic Fibrosis, 20(6), pp. 1010-1017.
184. Burtin, C., Van Remoortel, H., Vrijsen, B., Langer, D., Colpaert, K., Gosselink, R., Decramer, M., Dupont, L. and Troosters, T. (2013) 'Impact of exacerbations of cystic fibrosis on muscle strength', Respiratory Research, 14(1).
185. Clement, A., Tamalet, A., Leroux, E., Ravilly, S., Fauroux, B. and Jais, J. P. (2006) 'Long term effects of azithromycin in patients with cystic fibrosis: A double blind, placebo controlled trial', Thorax, 61(10), pp. 895-902.
186. Dill, E. J., Dawson, R., Sellers, D. E., Robinson, W. M. and Sawicki, G. S. (2013) 'Longitudinal trends in health-related quality of life in adults with cystic fibrosis', Chest, 144(3), pp. 981-989.
187. Elborn, J. S., Flume, P. A., Cohen, F., Loutit, J. and VanDevanter, D. R. (2016) 'Safety and efficacy of prolonged levofloxacin inhalation solution (APT-1026) treatment for cystic fibrosis and chronic Pseudomonas aeruginosa airway infection', Journal of Cystic Fibrosis, 15(5), pp. 634-640.
188. Escudero Garcia, J., Martin Rivada, A., Uribe Posada, A., Sanz Santiago, V., Argente, J. and Martos-Moreno, G. A. (2022) 'Carbohydrate metabolism impairment in children and adolescents with cystic fibrosis', Endocrinologia, Diabetes y Nutricion, 69(8), pp. 576-583.
189. Farhat, S. C. L., Almeida, M. B., Silva-Filho, L. V. R. F., Farhat, J., Rodrigues, J. C. and Braga, A. L. F. (2013) 'Ozone is associated with an increased risk of respiratory exacerbations in patients with cystic fibrosis', Chest, 144(4), pp. 1186-1192.
190. Conway, S. P., Pond, M. N., Watson, A., Etherington, C., Robey, H. L. and Goldman, M. H. (1997) 'Intravenous colistin sulphomethate in acute respiratory exacerbations in adult patients with cystic fibrosis', Thorax, 52(11), pp. 987-93.
191. Da Silva Filho, L. V. R. F., Zerbinati, R. M., Tateno, A. F., Boas, L. V., De Almeida, M. B., Levi, J. E., Drexler, J. F., Drosten, C. and Pannuti, C. S. (2012) 'The differential clinical impact of human coronavirus species in children with cystic fibrosis', Journal of Infectious Diseases, 206(3), pp. 384-388.
192. De Aquino Mota, C. S. B., Da Silva Filho, L. V. R. F. and Rodrigues, J. C. (2016) 'Impact of routine spirometry in the frequency of acute pulmonary exacerbation diagnosis in cystic fibrosis', Pediatric Pulmonology, 51(Supplement 42), pp. S50.
193. Safi, C., DiMango, E., Keating, C., Zhou, Z. and Gudis, D. A. (2020) 'Sinonasal quality-of-life declines in cystic fibrosis patients with pulmonary exacerbations', International forum of allergy & rhinology, 10(2), pp. 194-198.
194. Abu-Fraiha, Y., Elyashar-Earon, H., Shoseyov, D., Cohen-Cymberknoh, M., Armoni, S., Kerem, E. and Wilschanski, M. (2019) 'Increasing Vitamin D Serum Levels Is Associated With Reduced Pulmonary Exacerbations in Patients With Cystic Fibrosis', Journal of Pediatric Gastroenterology and Nutrition, 68(1), pp. 110-115.
195. Bailey, K. L., Murphy, P. J., Lineberry, O. K., Haack, M. R., Dickinson, J. D. and Kalil, A. C. (2020) 'Procalcitonin predicts the severity of cystic fibrosis pulmonary exacerbations and readmissions in adult patients: a prospective cohort study', Journal of investigative medicine : the official publication of the American Federation for Clinical Research, 68(4), pp. 856-863.
196. Burton, K., Morris, N. R., Reid, D., Smith, D. and Kuys, S. (2020) Increased physical activity post-exacerbation is associated with decreased systemic inflammation in cystic fibrosis – An observational study, Philadelphia, Pennsylvania: Taylor & Francis Ltd (0959-3985. Available at: https://search.ebscohost.com/login.aspx?direct=true&db=cin20&AN=146906644&site=ehost-live.

197. Erdmann, N., Schilling, T., Hentschel, J., Lehmann, T., von Bismarck, P., Ankermann, T., Duckstein, F., Baier, M., Zagoya, C. and Mainz, J. G. (2022) 'Divergent dynamics of inflammatory mediators and multiplex PCRs during airway infection in cystic fibrosis patients and healthy controls: Serial upper airway sampling by nasal lavage', Frontiers in Immunology, 13, pp. 947359.

198. Asner, S., Waters, V., Solomon, M., Yau, Y., Richardson, S. E., Grasemann, H., Gharabaghi, F. and Tran, D. (2012) 'Role of respiratory viruses in pulmonary exacerbations in children with cystic fibrosis', Journal of Cystic Fibrosis, 11(5), pp. 433-439.

199. McCoy, K. S., Quittner, A. L., Oermann, C. M., Gibson, R. L., Retsch-Bogart, G. Z. and Montgomery, A. B. (2008) 'Inhaled aztreonam lysine for chronic airway Pseudomonas aeruginosa in cystic fibrosis', American Journal of Respiratory and Critical Care Medicine, 178(9), pp. 921-8.

200. Wojsyk-Banaszak, I., Sobkowiak, P., Jonczyk-Potoczna, K., Narozna, B., Langwinski, W., Szczepanik, M., Kycler, Z., Breborowicz, A. and Szczepankiewicz, A. (2019) 'Evaluation of Copeptin during Pulmonary Exacerbation in Cystic Fibrosis', Mediators of Inflammation, 2019, pp. 1939740.

201. Aaron, S. D., Ramotar, K., Ferris, W., Vandemheen, K., Saginur, R., Tullis, E., Haase, D., Kottachchi, D., St. Denis, M. and Chan, F. (2004) 'Adult cystic fibrosis exacerbations and new strains of Pseudomonas aeruginosa', American Journal of Respiratory & Critical Care Medicine, 169(7), pp. 811-815.

202. Wainwright, C. E., Vidmar, S., Armstrong, D. S., Byrnes, C. A., Carlin, J. B., Cheney, J., Cooper, P. J., Grimwood, K., Moodie, M., Robertson, C. F. and Tiddens, H. A. (2011) 'Effect of bronchoalveolar lavage-directed therapy on Pseudomonas aeruginosa infection and structural lung injury in children with cystic fibrosis: a randomized trial', JAMA, 306(2), pp. 163-71.

203. Bradley, J., McAlister, O. and Elborn, S. (2001) 'Pulmonary function, inflammation, exercise capacity and quality of life in cystic fibrosis', The european respiratory journal, 17(4), pp. 712-5.

204. Yi, M. S., Britto, M. T., Wilmott, R. W., Kotagal, U. R., Eckman, M. H., Nielson, D. W., Kociela, V. L. and Tsevat, J. (2003) 'Health values of adolescents with cystic fibrosis', The Journal of Pediatrics, 142(2), pp. 133-40.

205. Sarfaraz, S., Sund, Z. and Jarad, N. (2010) 'Real-time, once-daily monitoring of symptoms and FEV in cystic fibrosis patients--a feasibility study using a novel device', The clinical respiratory journal, 4(2), pp. 74-82.

206. Srivastava, S. A., Burgess, J. C., Bilton, D., Hodson, M. E., Gyi, K. M. And Baker, E. H. (2010) 'Is HBA1C a useful marker for pulmonary exacerbations in patients with CF but no known diabetes?', Pediatric Pulmonology, 45(SUPPL. 33), pp. 375.

207. Ward, N., White, D., Rowe, H., Stiller, K. and Sullivan, T. (2013) 'Physical activity levels of patients with cystic fibrosis hospitalised with an acute respiratory exacerbation', Respiratory Medicine, 107(7), pp. 1014-20.

208. Perrem, L., Klingel, M., Stanojevic, S., Isaac, S. M., Jensen, R., Sanders, D. B., Solomon, M., Grasemann, H., Waters, V. J., Sweezey, N., Davis, S. D. And Ratjen, F. (2019) 'The lung clearance index can detect acute respiratory events in school-age children with cystic fibrosis', Pediatric Pulmonology, 54(Supplement 2), pp. 361.

209. Whitehead, A., Conway, S. P., Etherington, C., Caldwell, N. A., Setchfield, N. and Bogle, S. (2002) 'Once-daily tobramycin in the treatment of adult patients with cystic fibrosis', European Respiratory Journal, 19(2), pp. 303-309.

210. Perrem, L., Stanojevic, S., Klingel, M., Isaac, S., Jensen, R., Solomon, M., Grasemann, H., Waters, V., Sweezey, N., Davis, S. And Ratjen, F. (2019) 'Symptom duration and the lung clearance index in children with cystic fibrosis and pulmonary exacerbations', European Respiratory Journal, 54(Supplement 63).

211. Actrn (2020) 'Replacement of hospital clinic appointments with telehealth appointments delivered directly to the home for people with cystic fibrosis (CF)', https://trialsearch.who.int/Trial2.aspx?Trialid=ACTRN12620000311954.

212. Perrem, L., Stanojevic, S., Shaw, M., Jensen, R., Guido, J., Solomon, M., Grasemann, H., Sweezey, N., Davies, S., Sanders, D. B., mcdonald, N., Isaac, S., Jara, S., Clem, C. And Ratjen, F. (2020) 'The Lung Clearance Index detects incomplete lung function recovery with acute respiratory events in school-age children with cystic fibrosis', European Respiratory Journal, 56(Supplement 64).

213. Lobbes, H., Durupt, S., Mainbourg, S., Pereira, B., Nove-Josserand, R., Durieu, I. and Reynaud, Q. (2022) 'Iron Deficiency in Cystic Fibrosis: A Cross-Sectional Single-Centre Study in a Referral Adult Centre', Nutrients, 14(3).

214. Ali, H. A., Deraz, T. E., Mohamed, D. A. and Mahmoud, M. Z. (2022) 'Impact of vitamin D status on CF and non-CF bronchiectasis outcomes', Egyptian Pediatric Association Gazette, 70(1), pp. 3.

215. Altenburg, J., de Graaff, C. S., Stienstra, Y., Sloos, J. H., van Haren, E. H. J., Koppers, R. J. H., van der Werf, T. S. and Boersma, W. G. (2013) 'Effect of azithromycin maintenance treatment on infectious exacerbations among patients with non-cystic fibrosis bronchiectasis: the BAT randomized controlled trial', JAMA, 309(12), pp. 1251-9.

216. Blumer, J. L., Saiman, L., Konstan, M. W. and Melnick, D. (2005) 'The efficacy and safety of meropenem and tobramycin vs ceftazidime and tobramycin in the treatment of acute pulmonary exacerbations in patients with cystic fibrosis', Chest, 128(4), pp. 2336-2346.

217. Bucher, J., Boelle, P. Y., Hubert, D., Lebourgeois, M., Stremler, N., Durieu, I., Bremont, F., Deneuville, E., Delaisi, B., Corvol, H., Bassinet, L., Grenet, D., Remus, N., Vodoff, M. V., Boussaud, V., Troussier, F., Leruez-Ville, M., Treluyer, J. M., Launay, O. and Sermet-Gaudelus, I. (2016) 'Lessons from a French collaborative case-control study in cystic fibrosis patients during the 2009 A/H1N1 influenza pandemy', BMC Infectious Diseases, 16(1), pp. 55.

218. Chmiel, J. F., Flume, P., Downey, D. G., Dozor, A. J., Colombo, C., Mazurek, H., Sapiejka, E., Rachel, M., Constantine, S., Conley, B., Dgetluck, N., Dinh, Q., White, B. and Elborn, J. S. (2021) 'Safety and efficacy of lenabasum in a phase 2 randomized, placebo-controlled trial in adults with cystic fibrosis', Journal of cystic fibrosis : official journal of the European Cystic Fibrosis Society, 20(1), pp. 78-85.

219. Conway, S. P., Etherington, C., Munday, J., Goldman, M. H., Strong, J. J., Wootton, M., Conway, S. P., Etherington, C., Munday, J., Goldman, M. H., Strong, J. J. and Wootton, M. (2000) 'Safety and tolerability of bolus intravenous colistin in acute respiratory exacerbations in adults with cystic fibrosis', Annals of Pharmacotherapy, 34(11), pp. 1238-1242.

220. Devereux, G., Wrolstad, D., Bourke, S. J., Daines, C. L., Doe, S., Dougherty, R., Franco, R., Innes, A., Kopp, B. T., Lascano, J., Layish, D., MacGregor, G., Murray, L., Peckham, D., Lucidi, V., Lovie, E., Robertson, J., Fraser-Pitt, D. J. and O'Neil, D. A. (2020) 'Oral cysteamine as an adjunct treatment in cystic fibrosis pulmonary exacerbations: An exploratory randomized clinical trial', PLoS ONE, 15(12), pp. e0242945.

221. Dovey, M., Aitken, M. L., Emerson, J., McNamara, S., Waltz, D. A. and Gibson, R. L. (2007) 'Oral corticosteroid therapy in cystic fibrosis patients hospitalized for pulmonary exacerbation: a pilot study', Chest, 132(4), pp. 1212-8.

222. Flight, W. G., Bright-Thomas, R. J., Sarran, C., Mutton, K. J., Morris, J., Webb, A. K. and Jones, A. M. (2014) 'The effect of the weather on pulmonary exacerbations and viral infections among adults with cystic fibrosis', International Journal of Biometeorology, 58(9), pp. 1845-51.

223. Flight, W. G., Bright-Thomas, R. J., Tilston, P., Mutton, K. J., Guiver, M., Morris, J., Webb, A. K. and Jones, A. M. (2014) 'Incidence and clinical impact of respiratory viruses in adults with cystic fibrosis', Thorax, 69(3), pp. 247-53.

224. Gifford, A. H., Alexandru, D. M., Li, Z., Dorman, D. B., Moulton, L. A., Price, K. E., Hampton, T. H., Sogin, M. L., Zuckerman, J. B., Parker, H. W., Stanton, B. A. and O'Toole, G. A. (2014) 'Iron supplementation does not worsen respiratory health or alter the sputum microbiome in cystic fibrosis', Journal of Cystic Fibrosis, 13(3), pp. 311-318.

225. Green, H. D., Bright-Thomas, R. J., Mutton, K. J., Guiver, M. and Jones, A. M. (2016) 'Increased prevalence of Pneumocystis jirovecii colonisation in acute pulmonary exacerbations of cystic fibrosis', Journal of Infection, 73(1), pp. 1-7.

226. Hahn, A., Jensen, C., Fanous, H., Chaney, H., Sami, I., Perez, G. F., Louie, S., Koumbourlis, A. C., Bost, J. E. and van den Anker, J. N. (2018) 'Relationship of pulmonary outcomes, microbiology, and serum antibiotic concentrations in cystic fibrosis patients', Journal of Pediatric Pharmacology and Therapeutics, 23(5), pp. 379-389.

227. Hong, G., Alby, K., Ng, S. C. W., Fleck, V., Kubrak, C., Rubenstein, R. C., Dorgan, D. J., Kawut, S. M. and Hadjiliadis, D. (2020) 'The presence of Aspergillus fumigatus is associated with worse respiratory quality of life in cystic fibrosis', Journal of Cystic Fibrosis, 19(1), pp. 125-130.

228. Hubert, D., Le Roux, E., Lavrut, T., Wallaert, B., Scheid, P., Manach, D., Grenet, D., Sermet-Gaudelus, I., Ramel, S., Cracowski, C., Sardet, A., Wizla, N., Deneuville, E. and Garraffo, R. (2009) 'Continuous versus intermittent infusions of ceftazidime for treating exacerbation of cystic fibrosis', Antimicrobial Agents and Chemotherapy, 53(9), pp. 3650-3656.

229. Junge, S., Gorlich, D., den Reijer, M., Wiedemann, B., Tummler, B., Ellemunter, H., Dubbers, A., Kuster, P., Ballmann, M., Koerner-Rettberg, C., Grose-Onnebrink, J., Heuer, E., Sextro, W., Mainz, J. G., Hammermann, J., Riethmuller, J., Graepler-Mainka, U., Staab, D., Wollschlager, B., Szczepanski, R., Schuster, A., Tegtmeyer, F.-K., Sutharsan, S., Wald, A., Nofer, J.-R., van Wamel, W., Becker, K., Peters, G. and Kahl, B. C. (2016) 'Factors Associated with Worse Lung Function in Cystic Fibrosis Patients with Persistent Staphylococcus aureus', PLoS ONE, 11(11), pp. e0166220.

230. Al-Aloul, M., Nazareth, D. and Walshaw, M. (2019) 'The renoprotective effect of concomitant fosfomycin in the treatment of pulmonary exacerbations in cystic fibrosis', Clinical Kidney Journal, 12(5), pp. 652-658.

231. Aris, R. M., Stephens, A. R., Ontjes, D. A., Blackwood, A. D., Lark, R. K., Hensler, M. B., Neuringer, I. P. and Lester, G. E. (2000) 'Adverse alterations in bone metabolism are associated with lung infection in adults with cystic fibrosis', American Journal of Respiratory and Critical Care Medicine, 162(5), pp. 1674-1678.

232. Başaran, A. E., Başaran, A., Kocacik Uygun, D. F., Yılmaz, E., Moballegh, A., Öz, L., Alper, Ö. and Bingöl, A. (2021) 'Association Between Cystic Fibrosis Severity Markers and CFTR Genotypes in Turkish Children', Turkish Thoracic Journal / Turk Toraks Dergisi, 22(6), pp. 426-431.

233. Bell, S. C., Bowerman, A. M., Nixon, L. E., Macdonald, I. A., Elborn, J. S. and Shale, D. J. (2000) 'Metabolic and inflammatory responses to pulmonary exacerbation in adults with cystic fibrosis', European Journal of Clinical Investigation, 30(6), pp. 553-9.

234. Bensel, T., Stotz, M., Borneff-Lipp, M., Wollschlager, B., Wienke, A., Taccetti, G., Campana, S., Meyer, K. C., Jensen, P. O., Lechner, U., Ulrich, M., Doring, G. and Worlitzsch, D. (2011) 'Lactate in cystic fibrosis sputum', Journal of cystic fibrosis : official journal of the European Cystic Fibrosis Society, 10(1), pp. 37-44.

235. Billard-Pomares, T., Herwegh, S., Wizla-Derambure, N., Turck, D., Courcol, R. and Husson, M.-O. (2011) 'Application of quantitative PCR to the diagnosis and monitoring of Pseudomonas aeruginosa colonization in 5-18-year-old cystic fibrosis patients', Journal of Medical Microbiology, 60(Pt 2), pp. 157-161.

236. Bozzella, M. J., Chaney, H., Sami, I., Koumbourlis, A., Bost, J. E., Zemanick, E. T., Freishtat, R. J., Crandall, K. A. and Hahn, A. (2021) 'Impact of Anaerobic Antibacterial Spectrum on Cystic Fibrosis Airway Microbiome Diversity and Pulmonary Function', Pediatric Infectious Disease Journal, 40(11), pp. 962-968.

237. Brockbank, S., Downey, D., Elborn, J. S. and Ennis, M. (2005) 'Effect of cystic fibrosis exacerbations on neutrophil function', International Immunopharmacology, 5(3), pp. 601-608.

238. Burns, J. L., Emerson, J., Kuypers, J., Campbell, A. P., Gibson, R. L., McNamara, S., Worrell, K. and Englund, J. A. (2012) 'Respiratory viruses in children with cystic fibrosis: Viral detection and clinical findings', Influenza and other Respiratory Viruses, 6(3), pp. 218-223.

239. Cahen, P., Le Bourgeois, M., Delacourt, C., Coustere, C., Nicaise, P., de Blic, J., Veron, M., Scheinmann, P. and Gaillard, J. L. (1993) 'Serum bactericidal test as a prognostic indicator in acute pulmonary exacerbations of cystic fibrosis', Pediatrics, 91(2), pp. 451-5.

240. Castellani, S., D'Oria, S., Diana, A., Polizzi, A. M., Di Gioia, S., Mariggiò, M. A., Guerra, L., Favia, M., Vinella, A., Leonetti, G., De Venuto, D., Gallo, C., Montemurro, P. and Conese, M. (2019) 'G-CSF and GM-CSF Modify Neutrophil Functions at Concentrations found in Cystic Fibrosis', Scientific Reports, 9(1).

241. Chin, M., De Zoysa, M., Slinger, R., Gaudet, E., Vandemheen, K. L., Chan, F., Hyde, L., Mah, T.-F., Ferris, W., Mallick, R. and Aaron, S. D. (2015) 'Acute effects of viral respiratory tract infections on sputum bacterial density during CF pulmonary exacerbations', Journal of cystic fibrosis : official journal of the European Cystic Fibrosis Society, 14(4), pp. 482-9.

242. Chirico, V., Lacquaniti, A., Leonardi, S., Grasso, L., Rotolo, N., Romano, C., Di Dio, G., Lionetti, E., David, A., Arrigo, T., Salpietro, C. and La Rosa, M. (2015) 'Acute pulmonary exacerbation and lung function decline in patients with cystic fibrosis: High-mobility group box 1 (HMGB1) between inflammation and infection', Clinical Microbiology and Infection, 21(4), pp. 368.e1-368.e9.

243. Chiron, R., Grumbach, Y. Y., Quynh, N. V. T., Verriere, V. and Urbach, V. (2008) 'Lipoxin A4 and interleukin-8 levels in cystic fibrosis sputum after antibiotherapy', Journal of Cystic Fibrosis, 7(6), pp. 463-468.

244. Chotirmall, S. H., O'Donoghue, E., Bennett, K., Gunaratnam, C., O'Neill, S. J., McElvaney, N. G., Chotirmall, S. H., O'Donoghue, E., Bennett, K., Gunaratnam, C., O'Neill, S. J. and McElvaney, N. G. (2010) 'Sputum Candida albicans presages FEV_1 decline and hospital-treated exacerbations in cystic fibrosis', Chest, 138(5), pp. 1186-1195.

245. da Costa Capizzani, C. P., Cacador, N. C., Torres, L. A. G. M. M., Tonani, L., Vandamme, P. and da Costa Darini, A. L. (2017) 'Clinical and microbiological profile of chronic Burkholderia cepacia complex infections in a cystic fibrosis reference hospital in Brazil', European journal of clinical microbiology & infectious diseases : official publication of the European Society of Clinical Microbiology, 36(11), pp. 2263-2271.

246. Dales, L., Ferris, W., Vandemheen, K. and Aaron, S. D. (2009) 'Combination antibiotic susceptibility of biofilm-grown Burkholderia cepacia and Pseudomonas aeruginosa isolated from patients with pulmonary exacerbations of cystic fibrosis', European Journal of Clinical Microbiology and Infectious Diseases, 28(10), pp. 1275-1279.

247. de Almeida, M. B., Zerbinati, R. M., Tateno, A. F., Oliveira, C. M., Romão, R. M., Rodrigues, J. C., Pannuti, C. S. and da Silva Filho, L. V. F. (2010) 'Rhinovirus C and respiratory exacerbations in children with cystic fibrosis', Emerging Infectious Diseases, 16(6), pp. 996-999.

248. De Rose, V., Oliva, A., Messore, B., Grosso, B., Mollar, C. and Pozzi, E. (1998) 'Circulating adhesion molecules in cystic fibrosis', American Journal of Respiratory and Critical Care Medicine, 157(4 Pt 1), pp. 1234-9.

249. Eichler, I., Nilsson, M., Rath, R., Enander, I., Venge, P. and Koller, D. Y. (1999) 'Human neutrophil lipocalin, a highly specific marker for acute exacerbation in cystic fibrosis', The european respiratory journal, 14(5), pp. 1145-9.

250. Felton, E., Burrell, A., Chaney, H., Sami, I., Koumbourlis, A. C., Freishtat, R. J., Crandall, K. A. and Hahn, A. (2021) 'Inflammation in children with cystic fibrosis: contribution of bacterial production of long-chain fatty acids', Pediatric Research, 90(1), pp. 99-108.

251. Forrester, D. L., Barr, H. L., Fogarty, A. and Knox, A. (2017) 'sTREM-1 is elevated in cystic fibrosis and correlates with proteases', Pediatric Pulmonology, 52(4), pp. 467-471.

252. Foweraker, J. E., Laughton, C. R., Brown, D. F. and Bilton, D. (2009) 'Comparison of methods to test antibiotic combinations against heterogeneous populations of multiresistant Pseudomonas aeruginosa from patients with acute infective exacerbations in cystic fibrosis', Antimicrobial Agents and Chemotherapy, 53(11), pp. 4809-4815.

253. Frangolias, D. D., Ruan, J., Wilcox, P. J., Davidson, A. G. F., Wong, L. T. K., Berthiaume, Y., Hennessey, R., Freitag, A., Pedder, L., Corey, M., Sweezey, N., Zielenski, J., Tullis, E. and Sandford, A. J. (2003) 'Alpha 1-antitrypsin deficiency alleles in cystic fibrosis lung disease', American Journal of Respiratory Cell and Molecular Biology, 29(3 Pt 1), pp. 390-6.

254. Gaillard, J. L., Cahen, P., Delacourt, C., Silly, C., Le Bourgeois, M., Coustere, C., De Blic, J., Lenoir, G. and Scheinmann, P. (1995) 'Correlation between activity of Beta-Lactam agents in vitro and bacteriological outcome in acute pulmonary exacerbations of cystic fibrosis', European Journal of Clinical Microbiology and Infectious Diseases, 14(4), pp. 291-296.

255. Garcia-Nunez, M., Garcia-Gonzalez, M., Pomares, X., Monton, C., Millares, L., Quero, S., Prina, E., Asensio, O., Bosque, M., Capilla, S., Cuevas, O. and Monso, E. (2020) 'The Respiratory Microbiome in Cystic Fibrosis: Compartment Patterns and Clinical Relationships in Early Stage Disease', Frontiers in Microbiology, 11, pp. 1463.

256. Gifford, A. H., Moulton, L. A., Dorman, D. B., Olbina, G., Westerman, M., Parker, H. W., Stanton, B. A. and O'Toole, G. A. (2012) 'Iron homeostasis during cystic fibrosis pulmonary exacerbation', Clinical and Translational Science, 5(4), pp. 368-373.

257. Glikman, D., Siegel, J. D., David, M. Z., Okoro, N. M., Boyle-Vavra, S., Dowell, M. L. and Daum, R. S. (2008) 'Complex molecular epidemiology of methicillin-resistant Staphylococcus aureus isolates from children with cystic fibrosis in the era of epidemic community-associated methicillin-resistant S aureus', Chest, 133(6), pp. 1381-1387.

258. Hahn, A., Fanous, H., Jensen, C., Chaney, H., Sami, I., Perez, G. F., Koumbourlis, A. C., Louie, S., Bost, J. E., van den Anker, J. N., Freishtat, R. J., Zemanick, E. T. and Crandall, K. A. (2019) 'Changes in microbiome diversity following beta-lactam antibiotic treatment are associated with therapeutic versus subtherapeutic antibiotic exposure in cystic fibrosis', Scientific Reports, 9(1), pp. 2534.

259. Hanusch, B., Brinkmann, F., Mayorandan, S., Chobanyan-Jürgens, K., Wiemers, A., Jansen, K., Ballmann, M., Schmidt-Choudhury, A., Bollenbach, A., Derichs, N., Tsikas, D. and Lücke, T. (2020) 'Local and systemic alterations of the l-arginine/nitric oxide pathway in sputum, blood, and urine of pediatric cystic fibrosis patients and effects of antibiotic treatment', Journal of Clinical Medicine, 9(12), pp. 1-19.

260. Henke, M. O., John, G., Germann, M., Lindemann, H. and Rubin, B. K. (2007) 'MUC5AC and MUC5B mucins increase in cystic fibrosis airway secretions during pulmonary exacerbation', American Journal of Respiratory & Critical Care Medicine, 175(8), pp. 816-821.

261. Hoek, R. A. S., Paats, M. S., Pas, S. D., Bakker, M., Hoogsteden, H. C., Boucher, C. A. B. and Van Der Eerden, M. M. (2013) 'Incidence of viral respiratory pathogens causing exacerbations in adult cystic fibrosis patients', Scandinavian Journal of Infectious Diseases, 45(1), pp. 65-69.

262. Just, J., Moog-Lutz, C., Houzel-Charavel, A., Canteloup, S., Grimfeld, A., Witko-Sarsat, V. and Cayre, Y. E. (1999) 'Proteinase 3 mRNA expression is induced in monocytes but not in neutrophils of patients with cystic fibrosis', FEBS Letters, 457(3), pp. 437-440.

263. Kabra, S. K., Pawaiya, R., Lodha, R., Kapil, A., Kabra, M., Vani, A. S., Agarwal, G. and Shastri, S. S. (2010) 'Long-term daily high and low doses of azithromycin in children with cystic fibrosis: a randomized controlled trial', Journal of cystic fibrosis : official journal of the European Cystic Fibrosis Society, 9(1), pp. 17-23.

264. Khalid, S., McGrowder, D., Kemp, M. and Johnson, P. (2007) 'The use of soluble transferin receptor to assess iron deficiency in adults with cystic fibrosis', Clinica Chimica Acta, 378(1-2), pp. 194-200.

265. Kidd, J. M., Sakon, C. M., Oleksiuk, L. M., Cies, J. J., Pettit, R. S., Nicolau, D. P. and Kuti, J. L. (2020) 'Pharmacokinetics of telavancin in adult patients with cystic fibrosis during acute pulmonary exacerbation', Antimicrobial Agents and Chemotherapy, 64(1).

266. Kieninger, E., Singer, F., Tapparel, C., Alves, M. P., Latzin, P., Tan, H. L., Bossley, C., Casaulta, C., Bush, A., Davies, J. C., Kaiser, L. and Regamey, N. (2013) 'High rhinovirus burden in lower airways of children with cystic fibrosis', Chest, 143(3), pp. 782-790.

267. Koller, D. Y., Gotz, M., Wojnarowski, C. and Eichler, I. (1996) 'Relationship between disease severity and inflammatory markers in cystic fibrosis', Archives of Disease in Childhood, 75(6), pp. 498-501.

268. Kussek, P., Mesa, D., Vasconcelos, T. M., Rodrigues, L. S., Krul, D., Ibanez, H., Faoro, H., Palmeiro, J. K. and Costa, L. M. D. (2022) 'Lower airway microbiota and decreasing lung function in young Brazilian cystic fibrosis patients with pulmonary Staphylococcus and Pseudomonas infection', PLoS ONE, 17(8 August).

269. Lagrange-Puget, M., Durieu, I., Ecochard, R., Abbas-Chorfa, F., Drai, J., Steghens, J.-P., Pacheco, Y., Vital-Durand, D. and Bellon, G. (2004) 'Longitudinal study of oxidative status in 312 cystic fibrosis patients in stable state and during bronchial exacerbation', Pediatric Pulmonology, 38(1), pp. 43-9.

270. Layeghifard, M., Li, H., Wang, P. W., Donaldson, S. L., Coburn, B., Clark, S. T., Caballero, J. D., Zhang, Y., Tullis, D. E., Yau, Y. C. W., Waters, V., Hwang, D. M. and Guttman, D. S. (2019) 'Microbiome networks and change-point analysis reveal key community changes associated with cystic fibrosis pulmonary exacerbations', npj Biofilms and Microbiomes, 5(1), pp. 4.

271. Liou, T. G., Adler, F. R., Argel, N., Asfour, F., Brown, P. S., Chatfield, B. A., Daines, C. L., Durham, D., Francis, J. A., Glover, B., Heynekamp, T., Hoidal, J. R., Jensen, J. L., Keogh, R., Kopecky, C. M., Lechtzin, N., Li, Y., Lysinger, J., Molina, O., Nakamura, C., Packer, K. A., Poch, K. R., Quittner, A. L., Radford, P., Redway, A. J., Sagel, S. D., Sprandel, S., Taylor-Cousar, J. L., Vroom, J. B., Yoshikawa, R., Clancy, J. P., Elborn, J. S., Olivier, K. N. and Cox, D. R. (2019) 'Prospective multicenter randomized patient recruitment and sample collection to enable future measurements of sputum biomarkers of inflammation in an observational study of cystic fibrosis', BMC Medical Research Methodology, 19(1), pp. 88.

272. Master, V., Roberts, G. W., Coulthard, K. P., Baghurst, P. A., Martin, A., Roberts, M. E., Onishko, C. R., Martin, A. J., Linke, R. J., Holmes, M., Jarvinen, A., Kennedy, D., Colebatch, K. A., Hansman, D. and Parsons, D. W. (2001) 'Efficacy of once-daily tobramycin monotherapy for acute pulmonary exacerbations of cystic fibrosis: a preliminary study', Pediatric Pulmonology, 31(5), pp. 367-76.

273. McColley, S. A., Stellmach, V., Boas, S. R., Jain, M. and Crawford, S. E. (2000) 'Serum vascular endothelial growth factor is elevated in cystic fibrosis and decreases with treatment of acute pulmonary exacerbation', American Journal of Respiratory and Critical Care Medicine, 161(6), pp. 1877-1880.

274. McGrath, L. T., Patrick, R., Mallon, P., Dowey, L., Silke, B., Norwood, W. and Elborn, S. (2000) 'Breath isoprene during acute respiratory exacerbation in cystic fibrosis', The european respiratory journal, 16(6), pp. 1065-9.

275. McKiernan, P. J., Molloy, K. P., Glasgow, A. M. A., McElvaney, N. G. and Greene, C. M. (2021) 'miR-224-5p and miR-545-5p Levels Relate to Exacerbations and Lung Function in a Pilot Study of X-Linked MicroRNA Expression in Cystic Fibrosis Monocytes', Frontiers in Genetics, 12.

276. Mulcahy, E. M., Hudson, J. B., Beggs, S. A., Reid, D. W., Roddam, L. F. and Cooley, M. A. (2015) 'High peripheral blood th17 percent associated with poor lung function in cystic fibrosis', PLoS ONE, 10(3), pp. e0120912.

277. Neubauer, C., Kasi, A. S., Grahl, N., Sessions, A. L., Kopf, S. H., Kato, R., Hogan, D. A. and Newman, D. K. (2018) 'Refining the Application of Microbial Lipids as Tracers of Staphylococcus aureus Growth Rates in Cystic Fibrosis Sputum', Journal of Bacteriology, 200(24).

278. Nixon, L. S., Yung, B., Bell, S. C., Elborn, J. S. and Shale, D. J. (1998) 'Circulating immunoreactive interleukin-6 in cystic fibrosis', American Journal of Respiratory and Critical Care Medicine, 157(6 PART I), pp. 1764-1769.

279. Patel, D., Popple, S., Claydon, A., Modha, D. E. and Gaillard, E. A. (2020) 'Posaconazole therapy in children with cystic fibrosis and Aspergillus-related lung disease', Medical Mycology, 58(1), pp. 11-21.

280. Pettit, R. S., Neu, N., Cies, J. J., Lapin, C., Muhlebach, M. S., Novak, K. J., Nguyen, S. T., Saiman, L., Nicolau, D. P. and Kuti, J. L. (2016) 'Population pharmacokinetics of meropenem administered as a prolonged infusion in children with cystic fibrosis', Journal of Antimicrobial Chemotherapy, 71(1), pp. 189-195.

281. Pribble, C. G., Black, P. G., Bosso, J. A. and Turner, R. B. (1990) 'Clinical manifestations of exacerbations of cystic fibrosis associated with nonbacterial infections', The Journal of Pediatrics, 117(2 Pt 1), pp. 200-4.

282. Pukhalsky, A. L., Kapranov, N. I., Kalashnikova, E. A., Shmarina, G. V., Shabalova, L. A., Kokarovtseva, S. N., Pukhalskaya, D. A., Kashirskaja, N. J. and Simonova, O. I. (1999) 'Inflammatory markers in cystic fibrosis patients with lung Pseudomonas aeruginosa infection', Mediators of Inflammation, 8(3), pp. 159-167.

283. Raghuvanshi, R., Vasco, K., Vazquez-Baeza, Y., Jiang, L., Morton, J. T., Li, D., Gonzalez, A., DeRight Goldasich, L., Humphrey, G., Ackermann, G., Swafford, A. D., Conrad, D., Knight, R., Dorrestein, P. C. and Quinn, R. A. (2020) 'High-Resolution longitudinal dynamics of the cystic fibrosis sputum microbiome and metabolome through antibiotic therapy', mSystems, 5(3), pp. e00292-20.

284. Ratjen, F., Waters, V., Klingel, M., McDonald, N., Dell, S., Leahy, T. R., Yau, Y. and Grasemann, H. (2016) 'Changes in airway inflammation during pulmonary exacerbations in patients with cystic fibrosis and primary ciliary dyskinesia', The european respiratory journal, 47(3), pp. 829-36.

285. Reid, P. A., McAllister, D. A., Boyd, A. C., Innes, J. A., Porteous, D., Greening, A. P. and Gray, R. D. (2015) 'Measurement of serum calprotectin in stable patients predicts exacerbation and lung function decline in cystic fibrosis', American Journal of Respiratory & Critical Care Medicine, 191(2), pp. 233-236.

286. Roderfeld, M., Rath, T., Schulz, R., Seeger, W., Tschuschner, A., Graf, J. and Roeb, E. (2009) 'Serum matrix metalloproteinases in adult CF patients: Relation to pulmonary exacerbation', Journal of Cystic Fibrosis, 8(5), pp. 338-347.

287. Saavedra, M. T., Quon, B. S., Faino, A., Caceres, S. M., Poch, K. R., Sanders, L. A., Malcolm, K. C., Nichols, D. P., Sagel, S. D., Taylor-Cousar, J. L., Leach, S. M., Strand, M. and Nick, J. A. (2018) 'Whole Blood Gene Expression Profiling Predicts Severe Morbidity and Mortality in Cystic Fibrosis: A 5-Year Follow-Up Study', Annals of the American Thoracic Society, 15(5), pp. 589-598.

288. Sagwal, S., Chauhan, A., Kaur, J., Prasad, R., Singh, M. and Singh, M. (2020) 'Association of Serum TGF-beta1 Levels with Different Clinical Phenotypes of Cystic Fibrosis Exacerbation', Lung, 198(2), pp. 377-383.

289. Serisier, D. J., Carroll, M. P., Shute, J. K. and Young, S. A. (2009) 'Macrorheology of cystic fibrosis, chronic obstructive pulmonary disease & normal sputum', Respiratory Research, 10, pp. 63.

290. Smith, A. L., Doershuk, C., Goldmann, D., Gore, E., Hilman, B., Marks, M., Moss, R., Ramsey, B., Redding, G., Rubio, T., Williams-Warren, J., Wilmott, R., Wilson, H. D. and Yogev, R. (1999) 'Comparison of a beta-lactam alone versus beta-lactam and an aminoglycoside for pulmonary exacerbation in cystic fibrosis', The Journal of Pediatrics, 134(4), pp. 413-21.

291. St. Denis, M., Ramotar, K., Vandemheen, K., Tullis, E., Ferris, W., Chan, F., Lee, C., Slinger, R. and Aaron, S. D. (2007) 'Infection with Burkholderia cepacia complex bacteria and pulmonary exacerbations of cystic fibrosis', Chest, 131(4), pp. 1188-1196.

292. Thomas, S. R., Ray, A., Hodson, M. E. and Pitt, T. L. (2000) 'Increased sputum amino acid concentrations and auxotrophy of Pseudomonas aeruginosa in severe cystic fibrosis lung disease', Thorax, 55(9), pp. 795-7.

293. Tramper-Stranders, G. A., Wolfs, T. F. W., van Haren Noman, S., van Aalderen, W. M. C., Nagelkerke, A. F., Nuijsink, M., Kimpen, J. L. L. and van der Ent, C. K. (2010) 'Controlled trial of cycled antibiotic prophylaxis to prevent initial Pseudomonas aeruginosa infection in children with cystic fibrosis', Thorax, 65(10), pp. 915-20.

294. Vic, P., Ategbo, S., Turck, D., Husson, M. O., Launay, V., Loeuille, G. A., Sardet, A., Deschildre, A., Druon, D. and Arrouet-Lagande, C. (1998) 'Efficacy, tolerance, and pharmacokinetics of once daily tobramycin for pseudomonas exacerbations in cystic fibrosis', Archives of Disease in Childhood, 78(6), pp. 536-539.

295. Bodini, A., D'Orazio, C., Peroni, D. G., Corradi, M., Zerman, L., Folesani, G., Assael, B. M., Boner, A. L. and Piacentini, G. L. (2007) 'IL-8 and pH values in exhaled condensate after antibiotics in cystic fibrosis children', International Journal of Immunopathology and Pharmacology, 20(3), pp. 467-472.

296. Alicandro, G., Faelli, N., Gagliardini, R., Santini, B., Magazzu, G., Biffi, A., Rise, P., Galli, C., Tirelli, A. S., Loi, S., Valmarana, L., Cirilli, N., Palmas, T., Vieni, G., Bianchi, M. L., Agostoni, C. and Colombo, C. (2013) 'A randomized placebo-controlled study on high-dose oral algal docosahexaenoic acid supplementation in children with cystic fibrosis', Prostaglandins, leukotrienes, and essential fatty acids, 88(2), pp. 163-9.

297. Bal Topçu, D., Tugcu, G., Ozcan, F., Aslan, M., Yalcinkaya, A., Polat, S. E., Hizal, M., Yalcin, E. E., Ersoz, D. D., Ozcelik, U., Kiper, N., Lay, I. and Oztas, Y. (2020) 'Plasma Ceramides and Sphingomyelins of Pediatric Patients Increase in Primary Ciliary Dyskinesia but Decrease in Cystic Fibrosis', Lipids, 55(3), pp. 213-223.

298. Barbato, E., Daly, B., Douglas, S., Kerr, M., Litman, P. and Darrah, R. (2020) 'Genetic Variation Near chrXq22-q23 Is Linked to Emotional Functioning in Cystic Fibrosis', Biological Research for Nursing, 22(3), pp. 319-325.

299. Arvind, B., Medigeshi, G. R., Kapil, A., Xess, I., Singh, U., Lodha, R. and Kabra, S. K. (2020) 'Aetiological agents for pulmonary exacerbations in children with cystic fibrosis: An observational study from a tertiary care centre in northern India', The Indian journal of medical research, 151(1), pp. 65-70.

300. Richard, D. A., Nousia-Arvanitakis, S., Sollich, V., Hampel, B. J., Sommerauer, B. and Schaad, U. B. (1997) 'Oral ciprofloxacin vs. intravenous ceftazidime plus tobramycin in pediatric cystic fibrosis patients: comparison of antipseudomonas efficacy and assessment of safety with ultrasonography and magnetic resonance imaging. Cystic Fibrosis Study Group', The Pediatric infectious disease journal, 16(6), pp. 572-8.

301. Finnegan, M. J., Hughes, D. V. and Hodson, M. E. (1992) 'Comparison of nebulized and intravenous terbutaline during exacerbations of pulmonary infection in patients with cystic fibrosis', The European respiratory journal, 5(9), pp. 1089-91.

302. Hair, P. S., Sass, L. A., Vazifedan, T., Shah, T. A., Krishna, N. K. and Cunnion, K. M. (2017) 'Complement effectors, C5a and C3a, in cystic fibrosis lung fluid correlate with disease severity', PloS one, 12(3), pp. e0173257.

303. van Maarseveen, E. M., van der Meer, R., Neef, C., Heijerman, H. G. M. and Touw, D. J. (2020) 'Does Circadian Rhythm Affect the Pharmacokinetics of Once-Daily Tobramycin in Adults With Cystic Fibrosis?', Therapeutic drug monitoring, 42(4), pp. 595-599.

304. Straßburg, S., Linker, C.-M., Brato, S., Schöbel, C., Taube, C., Götze, J., Stehling, F., Sutharsan, S., Welsner, M. and Weinreich, G. (2022) 'Investigation of respiratory rate in patients with cystic fibrosis using a minimal-impact biomotion system', BMC Pulmonary Medicine, 22(1), pp. 1-6.

305. Pleasants, R. A., Michalets, E. L., Williams, D. M., Samuelson, W. M., Rehm, J. R. and Knowles, M. R. (1996) 'Pharmacokinetics of vancomycin in adult cystic fibrosis patients', Antimicrobial agents and chemotherapy, 40(1), pp. 186-90.

306. Montgomery, M.J., Beringer, P.M., Aminimanizani, A., Louie, S.G., Shapiro, B.J., Jelliffe, R. and Gill, M.A., 2001. Population pharmacokinetics and use of Monte Carlo simulation to evaluate currently recommended dosing regimens of ciprofloxacin in adult patients with cystic fibrosis. Antimicrobial agents and chemotherapy, 45(12), pp.3468-3473.

307. Dentice, R., Elkins, M. and Bye, P., 2015. A randomised controlled trial of hypertonic saline inhalation to enhance airway clearance physiotherapy in adults hospitalised with Cystic Fibrosis (CF). Physiotherapy, 101, p.e356.

308. de Boer, K., Vandemheen, K.L., Tullis, E., Doucette, S., Fergusson, D., Freitag, A., Paterson, N., Jackson, M., Lougheed, M.D., Kumar, V. and Aaron, S.D., 2011. Relationship Between Exacerbation Frequency And Clinical Outcomes In Patients With Cystic Fibrosis. A23. ADULT CYSTIC FIBROSIS: ADVANCES IN TREATMENT AND UNDERSTANDING OF MECHANISMS OF DISEASE, pp.A1113-A1113.

309. Rath, T., Zwaschka, L., Hage, L., Kugler, M., Menendez, K., Naehrlich, L., Schulz, R., Roderfeld, M. and Roeb, E. (2014) 'Identification of neutrophil activation markers as novel surrogate markers of CF lung disease', PLoS ONE [Electronic Resource], 9(12), p. e115847.

310. Hoen, A.G., Li, J., Moulton, L.A., O'Toole, G.A., Housman, M.L., Koestler, D.C., Guill, M.F., Moore, J.H., Hibberd, P.L., Morrison, H.G., Sogin, M.L., Karagas, M.R. and Madan, J.C. (2015) 'Associations between Gut Microbial Colonization in Early Life and Respiratory Outcomes in Cystic Fibrosis', Journal of Pediatrics, 167(1), pp. 138-147.e3.

311. Wood, L. G., Fitzgerald, D. A., Gibson, P. G., Cooper, D. M. and Garg, M. L. (2002) 'Increased plasma fatty acid concentrations after respiratory exacerbations are associated with elevated oxidative stress in cystic fibrosis patients', American Journal of Clinical Nutrition, 75(4), pp. 668-675.

312. Bradley, J. M., Koker, P., Deng, Q., Moroni-Zentgraf, P., Ratjen, F., Geller, D. E. and Stuart Elborn, J. (2014) 'Testing two different doses of tiotropium Respimat® in cystic fibrosis: Phase 2 randomized trial results', PloS one, 9(9).

313. Cantin, A. M., Bilodeau, G., Larivee, P. and Richter, M. V. (2012) 'Plasma biomarkers and cystic brosis fibrosis lung disease', Clinical and Investigative Medicine, 35(4), pp. E173-E181.

314. Carter, S. C., Franciosi, A. N., O'Shea, K. M., O'Carroll, O. M., Sharma, A., Bell, A., Keogan, B., O'Reilly, P., Coughlan, S., Law, S. M., Gray, R. D., Hisert, K. B., Singh, P. K., Cooke, G., Grogan, B., De Gascun, C. F., Gallagher, C. G., Nicholson, T. T., Quon, B. S. and McKone, E. F. (2022) 'Acute Pulmonary Exacerbation Phenotypes in Patients with Cystic Fibrosis', Annals of the American Thoracic Society, 19(11), pp. 1818-1826.

315. Chatham, K., Ionescu, A. A., Nixon, L. S. and Shale, D. J. (2004) 'A short-term comparison of two methods of sputum expectoration in cystic fibrosis', The European respiratory journal, 23(3), pp. 435-9.

316. Chotirmall, S. H., Branagan, P., Gunaratnam, C. and McElvaney, N. G. (2008) 'Aspergillus/allergic bronchopulmonary aspergillosis in an Irish cystic fibrosis population: a diagnostically challenging entity', Respiratory Care, 53(8), pp. 1035-41.

317. Cunningham, S., McColm, J. R., Mallinson, A., Boyd, I. and Marshall, T. G. (2003) 'Duration of effect of intravenous antibiotics on spirometry and sputum cytokines in children with cystic fibrosis', Pediatric pulmonology, 36(1), pp. 43-8.

318. De Boeck, K., Munck, A., Walker, S., Faro, A., Hiatt, P., Gilmartin, G. and Higgins, M. (2014) 'Efficacy and safety of ivacaftor in patients with cystic fibrosis and a non-G551D gating mutation', Journal of Cystic Fibrosis, 13(6), pp. 674-680.

319. Di Nardo, G., Oliva, S., Menichella, A., Pistelli, R., De Biase, R. V., Patriarchi, F., Cucchiara, S. and Stronati, L. (2014) 'Lactobacillus reuteri ATCC55730 in cystic fibrosis', Journal of pediatric gastroenterology and nutrition, 58(1), pp. 81-6.

320. Dogru, D., Dalgic, F., Kiper, N., Ozcelik, U., Yalcin, E., Aslan, A. T., Gurcan, N., Saricaoglu, F., Gur, D., Karayazgan, Y. and Firat, P. (2009) 'Long-term clarithromycin in cystic fibrosis: effects on inflammatory markers in BAL and clinical status', The Turkish journal of pediatrics, 51(5), pp. 416-23.

321. Duggan, C., Colin, A. A., Agil, A., Higgins, L. and Rifai, N. (1996) 'Vitamin A status in acute exacerbations of cystic fibrosis', American Journal of Clinical Nutrition, 64(4), pp. 635-639.

322. Esmond, G., Butler, M. and McCormack, A. M. (2006) 'Comparison of hospital and home intravenous antibiotic therapy in adults with cystic fibrosis', Journal of Clinical Nursing (Wiley-Blackwell), 15(1), pp. 52-60.

323. Fodor, A. A., Klem, E. R., Gilpin, D. F., Elborn, J. S., Boucher, R. C., Tunney, M. M. and Wolfgang, M. C. (2012) 'The adult cystic fibrosis airway microbiota is stable over time and infection type, and highly resilient to antibiotic treatment of exacerbations', PloS one, 7(9), pp. e45001.

324. Girón-Moreno, R. M., Justicia, J. L., Yamamoto, S., Valenzuela, C., Cisneros, C., Gómez-Punter, R. M., Fernandes-Vasconcelos, G. and Ancochea, J. (2014) 'Role of C-reactive protein as a biomarker for prediction of the severity of pulmonary exacerbations in patients with cystic fibrosis', BMC Pulmonary Medicine, 14(1), pp. 150-150.

325. Gray, R. D., Imrie, M., Boyd, A. C., Porteous, D., Innes, J. A. and Greening, A. P. (2010) 'Sputum and serum calprotectin are useful biomarkers during CF exacerbation', Journal of cystic fibrosis : official journal of the European Cystic Fibrosis Society, 9(3), pp. 193-8.

326. Hahn, A., Burrell, A., Chaney, H., Sami, I., Koumbourlis, A. C., Freishtat, R. J., Zemanick, E. T., Louie, S. and Crandall, K. A. (2021) 'Importance of beta-lactam pharmacokinetics and pharmacodynamics on the recovery of microbial diversity in the airway of persons with cystic fibrosis', Journal of investigative medicine : the official publication of the American Federation for Clinical Research, 69(7), pp. 1350-1359.

327. Hardy, S., Berardis, S., Aubriot, A. S., Reychler, G. and Gohy, S. (2022) 'One-minute sit-to-stand test is practical to assess and follow the muscle weakness in cystic fibrosis', Respiratory Research, 23(1).

328. Harris, W. T., Muhlebach, M. S., Oster, R. A., Knowles, M. R., Clancy, J. P. and Noah, T. L. (2011) 'Plasma TGF-beta1 in pediatric cystic fibrosis: potential biomarker of lung disease and response to therapy', Pediatric pulmonology, 46(7), pp. 688-95.

329. Heltshe, S. L., Saiman, L., Popowitch, E. B., Miller, M. B., Kloster, M., Thompson, V., Ferkol, T. W., Hoover, W. C., Schechter, M. S. and Muhlebach, M. S. (2015) 'Outcomes and Treatment of Chronic Methicillin-Resistant Staphylococcus aureus Differs by Staphylococcal Cassette Chromosome mec (SCCmec) Type in Children With Cystic Fibrosis', Journal of the Pediatric Infectious Diseases Society, 4(3), pp. 225-31.

330. Hendry, J., Elborn, J. S., Nixon, L., Shale, D. J. and Webb, A. K. (1999) 'Cystic fibrosis: inflammatory response to infection with Burkholderia cepacia and Pseudomonas aeruginosa', The European respiratory journal, 14(2), pp. 435-8.

331. Horak, F., Jr., Moeller, A., Singer, F., Straub, D., Holler, B., Helbich, T. H., Schneider, B., Eichler, I., Wildhaber, J. H. and Hall, G. L. (2007) 'Longitudinal monitoring of pediatric cystic fibrosis lung disease using nitrite in exhaled breath condensate', Pediatric pulmonology, 42(12), pp. 1198-206.

332. Ionescu, A. A., Nixon, L. S., Evans, W. D., Stone, M. D., Lewis-Jenkins, V., Chatham, K. and Shale, D. J. (2000) 'Bone density, body composition, and inflammatory status in cystic fibrosis', American journal of respiratory and critical care medicine, 162(3 Pt 1), pp. 789-94.

333. Klingel, M., Stanojevic, S., Tullis, E., Ratjen, F. and Waters, V. (2019) 'Oral Azithromycin and Response to Pulmonary Exacerbations Treated with Intravenous Tobramycin in Children with Cystic Fibrosis', Annals of the American Thoracic Society, 16(7), pp. 861-867.

334. Laguna, T. A., Wagner, B. D., Luckey, H. K., Mann, S. A., Sagel, S. D., Regelmann, W., Accurso, F. J., Laguna, T. A., Wagner, B. D., Luckey, H. K., Mann, S. A., Sagel, S. D., Regelmann, W. and Accurso, F. J. (2009) 'Sputum desmosine during hospital admission for pulmonary exacerbation in cystic fibrosis', CHEST, 136(6), pp. 1561-1568.

335. Laguna, T. A., Wagner, B. D., Starcher, B., Luckey Tarro, H. K., Mann, S. A., Sagel, S. D. and Accurso, F. J. (2012) 'Urinary desmosine: a biomarker of structural lung injury during CF pulmonary exacerbation', Pediatric pulmonology, 47(9), pp. 856-63.

336. Laguna, T. A., Williams, C. B., Brandy, K. R., Welchlin-Bradford, C., Moen, C. E., Reilly, C. S. and Wendt, C. H. (2015) 'Sputum club cell protein concentration is associated with pulmonary exacerbation in cystic fibrosis', Journal of cystic fibrosis : official journal of the European Cystic Fibrosis Society, 14(3), pp. 334-40.

337. Lindley, B., Bhakta, Z., Gray, K., Watanabe, A., Leclair, L. and Young, D. C. (2022) 'Pharmacokinetics of intermittent dosed intravenous vancomycin in adult persons with cystic fibrosis', Pediatric pulmonology, 57(11), pp. 2646-2651.

338. Mizutani, M., Berube, J., Ahlgren, H. G., Bernier, J., Matouk, E., Nguyen, D. and Rousseau, S. (2017) 'Corticosteroid-resistant inflammatory signalling in pseudomonas-infected bronchial cells', ERJ Open Research, 3(2), pp. 00144-2016.

339. Montemurro, P., Mariggio, M. A., Barbuti, G., Cassano, A., Vincenti, A., Serio, G., Guerra, L., Diana, A., Santostasi, T., Polizzi, A., Fumarulo, R., Casavola, V., Manca, A. and Conese, M. (2012) 'Increase in interleukin-8 production from circulating neutrophils upon antibiotic therapy in cystic fibrosis patients', Journal of cystic fibrosis : official journal of the European Cystic Fibrosis Society, 11(6), pp. 518-24.

340. Norman, D., Elborn, J. S., Cordon, S. M., Rayner, R. J., Wiseman, M. S., Hiller, E. J. and Shale, D. J. (1991) 'Plasma tumour necrosis factor alpha in cystic fibrosis', Thorax, 46(2), pp. 91-5.

341. Paats, M.S., Bergen, I.M., Bakker, M., Hoek, R.A., Nietzman-Lammering, K.J., Hoogsteden, H.C., Hendriks, R.W. and van der Eerden, M.M., 2013. Cytokines in nasal lavages and plasma and their correlation with clinical parameters in cystic fibrosis. Journal of Cystic Fibrosis, 12(6), pp.623-629.

342. Punch, G., Syrmis, M.W., Rose, B.R. et al. Method for detection of respiratory viruses in the sputa of patients with cystic fibrosis. Eur J Clin Microbiol Infect Dis 24, 54–57 (2005). https://doi.org/10.1007/s10096-004-1273-7

343. Reeves, E. P., Bergin, D. A., Fitzgerald, S., Hayes, E., Keenan, J., Henry, M., Meleady, P., Vega-Carrascal, I., Murray, M. A., Low, T. B., McCarthy, C., O'Brien, E., Clynes, M., Gunaratnam, C. and McElvaney, N. G. (2012) 'A novel neutrophil derived inflammatory biomarker of pulmonary exacerbation in cystic fibrosis', Journal of cystic fibrosis : official journal of the European Cystic Fibrosis Society, 11(2), pp. 100-7.

344. Regamey, N., Tsartsali, L., Hilliard, T. N., Fuchs, O., Tan, H.-L., Zhu, J., Qiu, Y.-S., Alton, E. W. F. W., Jeffery, P. K., Bush, A. and Davies, J. C. (2012) 'Distinct patterns of inflammation in the airway lumen and bronchial mucosa of children with cystic fibrosis', Thorax, 67(2), pp. 164-70.

345. Reilly, C. C., Jolley, C. J., Elston, C., Moxham, J. and Rafferty, G. F. (2012) 'Measurement of parasternal intercostal electromyogram during an infective exacerbation in patients with cystic fibrosis', The European respiratory journal, 40(4), pp. 977-81.

346. Renner, S., Rath, R., Rust, P., Lehr, S., Frischer, T., Elmadfa, I. and Eichler, I. (2001) 'Effects of beta-carotene supplementation for six months on clinical and laboratory parameters in patients with cystic fibrosis', Thorax, 56(1), pp. 48-52.

347. Robinson, T. E., Leung, A. N., Northway, W. H., Blankenberg, F. G., Bloch, D. A., Oehlert, J. W., Al-Dabbagh, H., Hubli, S. and Moss, R. B. (2001) 'Spirometer-triggered high-resolution computed tomography and pulmonary function measurements during an acute exacerbation in patients with cystic fibrosis', Journal of Pediatrics, 138(4), pp. 553-559.

348. Sagel, S. D., Thompson, V., Chmiel, J. F., Montgomery, G. S., Nasr, S. Z., Perkett, E., Saavedra, M. T., Slovis, B., Anthony, M. M., Emmett, P. and Heltshe, S. L. (2015) 'Effect of treatment of cystic fibrosis pulmonary exacerbations on systemic inflammation', Annals of the American Thoracic Society, 12(5), pp. 708-717.

349. Sharma, G., Lodha, R., Shastri, S., Saini, S., Kapil, A., Singla, M., Mukherjee, A., Jat, K. R., Kabra, M. and Kabra, S. K. (2016) 'Zinc Supplementation for One Year Among Children with Cystic Fibrosis Does Not Decrease Pulmonary Infection', Respiratory Care, 61(1), pp. 78-84.

350. Shead, E. F., Haworth, C. S., Gunn, E., Bilton, D., Scott, M. A. and Compston, J. E. (2006) 'Osteoclastogenesis during infective exacerbations in patients with cystic fibrosis', American Journal of Respiratory & Critical Care Medicine, 174(3), pp. 306-311.

351. Smith, D. J., Badrick, A. C., Zakrzewski, M., Krause, L., Bell, S. C., Anderson, G. J. and Reid, D. W. (2014) 'Pyrosequencing reveals transient cystic fibrosis lung microbiome changes with intravenous antibiotics', The European respiratory journal, 44(4), pp. 922-30.

352. Soret, P., Vandenborght, L.-E., Francis, F., Coron, N., Enaud, R., Avalos, M., Schaeverbeke, T., Berger, P., Fayon, M., Thiebaut, R. and Delhaes, L. (2020) 'Respiratory mycobiome and suggestion of inter-kingdom network during acute pulmonary exacerbation in cystic fibrosis', Scientific reports, 10(1), pp. 3589.

353. Srivastava, S. A., Nguyen, T. T., Burgess, J. C., Bilton, D., Hodson, M. E., Gyi, K. M. and Baker, E. H. (2010) 'Breath glucose as a marker of pulmonary inflammation in adult patients with cystic fibrosis', Pediatric pulmonology, 45(SUPPL. 33), pp. 375.

354. Stachowiak, Z., Wojsyk-Banaszak, I., Jonczyk-Potoczna, K., Narozna, B., Langwinski, W., Kycler, Z., Sobkowiak, P., Breborowicz, A. and Szczepankiewicz, A. (2020) 'Mirna expression profile in the airways is altered during pulmonary exacerbation in children with cystic fibrosis-A preliminary report', Journal of Clinical Medicine, 9(6), pp. 1-11.

355. Stephen, M. J., Hadjiliadis, D., Hoag, J. B., Holsclaw, D., Fiel, S., Varlotta, L., Zanni, R. L. and Bonsall, C. (2015) 'Daily home spirometry use in CF patients with an acute exacerbation', Pediatric pulmonology, 50(SUPPL. 41), pp. 357-358.

356. Tai, A. S., Sherrard, L. J., Kidd, T. J., Ramsay, K. A., Buckley, C., Syrmis, M., Grimwood, K., Bell, S. C. and Whiley, D. M. (2017) 'Antibiotic perturbation of mixed-strain Pseudomonas aeruginosa infection in patients with cystic fibrosis', BMC Pulmonary Medicine, 17, pp. 1-10.

357. Tingpej, P., Elkins, M., Rose, B., Hu, H., Moriarty, C., Manos, J., Barras, B., Bye, P. and Harbour, C. (2010) 'Clinical profile of adult cystic fibrosis patients with frequent epidemic clones of Pseudomonas aeruginosa', Respirology (Carlton, Vic.), 15(6), pp. 923-9.

358. Tirelli, A. S., Colombo, C., Torresani, E., Fortunato, F., Biffi, A., Cariani, L., Dacco, V., Carbone, A., Edefonti, A., Paglialonga, F. and Conese, M. (2013) 'Effects of treatment in the levels of circulating cytokines and growth factors in cystic fibrosis and dialyzed patients by multi-analytical determination with a biochip array platform', Cytokine, 62(3), pp. 413-20.

359. Topcu, D. B., Tugcu, G., Er, B., Polat, S. E., Hizal, M., Yalcin, E. E., Ersoz, D. D., Coplu, L., Ozcelik, U., Kiper, N., Lay, I. and Oztas, Y. (2022) 'Increased Plasma YKL-40 Level and Chitotriosidase Activity in Cystic Fibrosis Patients', Inflammation, 45(2), pp. 627-638.

360. Tunney, M. M., Klem, E. R., Fodor, A. A., Gilpin, D. F., Moriarty, T. F., McGrath, S. J., Muhlebach, M. S., Boucher, R. C., Cardwell, C., Doering, G., Elborn, J. S. and Wolfgang, M. C. (2011) 'Use of culture and molecular analysis to determine the effect of antibiotic treatment on microbial community diversity and abundance during exacerbation in patients with cystic fibrosis', Thorax, 66(7), pp. 579-584.

361. van Horck, M., Alonso, A., Wesseling, G., de Winter-de Groot, K., van Aalderen, W., Hendriks, H., Winkens, B., Rijkers, G., Jobsis, Q. and Dompeling, E. (2016) 'Biomarkers in Exhaled Breath Condensate Are Not Predictive for Pulmonary Exacerbations in Children with Cystic Fibrosis: Results of a One-Year Observational Study', PloS one, 11(4), pp. e0152156.

362. Van Wye, J. E., Collins, M. S., Baylor, M., Pennington, J. E., Hsu, Y. P., Sampanvejsopa, V. and Moss, R. B. (1990) 'Pseudomonas hyperimmune globulin passive immunotherapy for pulmonary exacerbations in cystic fibrosis', Pediatric pulmonology, 9(1), pp. 7-18.

363. Vanderhelst, E., De Meirleir, L., Schuermans, D., Malfroot, A., Vincken, W. and Verbanck, S. (2014) 'Evidence of an Acinar Response following Treatment for Exacerbation in Adult Patients with Cystic Fibrosis', Respiration, 87(6), pp. 492-498.

364. Vic, P., Ategbo, S., Gottrand, F., Launay, V., Loeuille, G. A., Elian, J. C., Druon, D., Farriaux, J. P., Turck, D., Vic, P., Ategbo, S., Gottrand, F., Launay, V., Loeuille, G. A., Elian, J. C., Druon, D., Farriaux, J. P. and Turck, D. (1997) 'Nutritional impact of antipseudomonas intravenous antibiotic courses in cystic fibrosis', Archives of Disease in Childhood, 76(5), pp. 437-440.

365. Voldby, C., Green, K., Kongstad, T., Ring, A. M., Sandvik, R. M., Skov, M., Buchvald, F., Pressler, T. and Nielsen, K. G. (2020) 'Lung clearance index-triggered intervention in children with cystic fibrosis – A randomised pilot study', Journal of Cystic Fibrosis, 19(6), pp. 934-941.

366. Wat, D., Gelder, C., Hibbitts, S., Cafferty, F., Bowler, I., Pierrepoint, M., Evans, R. and Doull, I. (2008) 'The role of respiratory viruses in cystic fibrosis', Journal of cystic fibrosis : official journal of the European Cystic Fibrosis Society, 7(4), pp. 320-328.

367. Wieneke, M. K., Dach, F., Neumann, C., Gorlich, D., Kaese, L., Thisen, T., Dubbers, A., Kessler, C., Grose-Onnebrink, J., Kuster, P., Schultingkemper, H., Schwartbeck, B., Roth, J., Nofer, J.-R., Treffon, J., Posdorfer, J., Boecken, J. M., Strake, M., Abdo, M., Westhues, S. and Kahl, B. C. (2021) 'Association of Diverse Staphylococcus aureus Populations with Pseudomonas aeruginosa Coinfection and Inflammation in Cystic Fibrosis Airway Infection', mSphere, 6(3), pp. e0035821.

368. Wojnarowski, C., Frischer, T., Hofbauer, E., Grabner, C., Mosgoeller, W., Eichler, I. and Ziesche, R. (1999) 'Cytokine expression in bronchial biopsies of cystic fibrosis patients with and without acute exacerbation', The European respiratory journal, 14(5), pp. 1136-44.

369. Wolter, J. M., Bowler, S. D. and McCormack, J. G. (1999) 'Are antipseudomonal antibiotics really beneficial in acute respiratory exacerbations of cystic fibrosis?', Australian and New Zealand journal of medicine, 29(1), pp. 15-21.

370. Wolter, J. M., Bowler, S. D., Nolan, P. J. and McCormack, J. G. (1997) 'Home intravenous therapy in cystic fibrosis: a prospective randomized trial examining clinical, quality of life and cost aspects', The European respiratory journal, 10(4), pp. 896-900.

371. Wolter, J. M., Rodwell, R. L., Bowler, S. D. and McCormack, J. G. (1999) 'Cytokines and inflammatory mediators do not indicate acute infection in cystic fibrosis', Clinical and diagnostic laboratory immunology, 6(2), pp. 260-5.

372. Woollam, M., Siegel, A. P., Grocki, P., Saunders, J. L., Sanders, D. B., Agarwal, M. and Davis, M. D. (2022) 'Preliminary method for profiling volatile organic compounds in breath that correlate with pulmonary function and other clinical traits of subjects diagnosed with cystic fibrosis: a pilot study', Journal of breath research, 16(2).

373. Worlitzsch, D., Rintelen, C., Bohm, K., Wollschlager, B., Merkel, N., Borneff-Lipp, M. and Doring, G. (2009) 'Antibiotic-resistant obligate anaerobes during exacerbations of cystic fibrosis patients', Clinical microbiology and infection : the official publication of the European Society of Clinical Microbiology and Infectious Diseases, 15(5), pp. 454-60.

374. Xu, X., Abdalla, T., Bratcher, P. E., Jackson, P. L., Sabbatini, G., Wells, J. M., Lou, X.-Y., Quinn, R., Blalock, J. E., Clancy, J. P. and Gaggar, A. (2017) 'Doxycycline improves clinical outcomes during cystic fibrosis exacerbations', The European respiratory journal, 49(4).

375. Yasar, P. A., Kose, M., Erdem, S., Hangul, M., Karaman, Z. F. and Eken, A. (2022) 'Circulating fibrocyte level in children with cystic fibrosis', Pediatrics international : official journal of the Japan Pediatric Society, 64(1), pp. e15058.

376. Arens, R., Gozal, D., Omlin, K.J., Vega, J., Boyd, K.P., Keens, T.G. and Woo, M.S., 1994. Comparison of high frequency chest compression and conventional chest physiotherapy in hospitalized patients with cystic fibrosis. American journal of respiratory and critical care medicine, 150(4), pp.1154-1157.

377. Strateva, T., Abrashev, I., Eneva, R., Petrova, G. and Mitov, I. (2009) 'Neuraminidases produced by cystic fibrosis isolates of Pseudomonas aeruginosa: Gene distribution, enzyme activity and clinical importance', Problems of Infectious and Parasitic Diseases, 37(2), pp. 25-27.

Full definitions of the commonly used PEx definitions.

Name of the definition	AGE	Criteria
Fuchs definition (1994)<br>Or<br>The DNase trial (1)	> 5 years	four or more of the following 12 symptoms or signs were considered to have a pulmonary exacerbation and were treated with parenteral antibiotics:<br>1. change in sputum.<br>2. New or increased hemoptysis.<br>3. Increased cough.<br>4. Increased dyspnea.<br>5. Malesia, fatigue, or lethargy.<br>6. Temperature above 38 C.<br>7. Anorexia or weight loss.<br>8. Sinus pain or tenderness.<br>9. Change in sinus discharge.<br>10. Change in physical examination of the chest.<br>11. Decreased in pulmonary function by %10 or more from previous recorded value.<br>12. Radiographic changes indicative of pulmonary infection
EuroCareCF Working Group or The European Consensus Group (2)<br>.		An exacerbation will be defined as the need for additional antibiotic treatment as indicated by a recent change in at least two of the following:<br>1. Change in sputum volume or colour<br>2. Increased cough<br>3. Increased malaise, fatigue or lethargy<br>4. Anorexia or weight loss<br>5. Decrease in pulmonary function by 10% or more /Radiographic changes<br>6. Increased dyspnoea

EPIC study (2009) (3)	1–12 year	The presence of a pulmonary exacerbation is established by th following: One of the major criteria alone or two of the minor signs/symptoms and fulfillment of symptom duration.<br>Major criteria: (one finding alone establishes the presence of a pulmonary exacerbation)<br>1. Decrease in FEV_1 of ≥10% from best baseline within past months, unresponsive to albuterol (in participants able to reproducibly perform spirometry)<br>2. Oxygen saturation b90% on room air or ≥5% decline from previous baseline<br>3. New lobar infiltrate(s) or atelectasi(e)s on chest radiograp<br>4. Hemoptysis (more than streaks on more than one occasio in past week)<br>Minor signs/symptoms: (two minor signs/symptoms are requir with duration criteria in the absence of major criteria)<br>1. Increased work of breathing or respiratory rate<br>2. New or increased adventitial sounds on lung exam<br>3. Weight loss ≥5% of body weight or decrease across 1 major percentile in weight percentile for age in past 6 months<br>4. Increased cough<br>5. Decreased exercise tolerance or level of activity<br>6. Increased chest congestion or change in sputum<br>Signs/symptom duration: (required with two minor signs/symptoms in absence of major criteria)<br>1. Duration of sign/symptoms ≥5 days or significant symptom severity.
US CF Foundation (CFF) (4)	> 10 years	A PE is defined as the presence of a least three of the following 11 new findings or changes in clinical status when compared to the most recent baseline visit. If the patient has not been seen within the previous three months, “baseline pulmonary status” must be judged from the patient’s own perception of well-being.<br>1. Increased cough<br>2. Increased sputum production and/or a change in appearance of expectorated sputum<br>3. Fever (>38 C for at least four hours in a 24-hour period) on more than one occasion in the previous week<br>4. Weight loss > 1 kg or 5% of body weight associated with anorexia and decreased dietary intake or growth failure in an infant or child<br>5. School or work absenteeism (due to illness) in the previous week<br>6. Increased respiratory rate and/or work of breathing<br>7. New findings on chest examination (e.g., rales, wheezing, crackles)

		8. Decreased exercise tolerance<br>9. Decrease in Forced Expiratory Volume at one second (FEV1) of > 10% from previous baseline study within past three months<br>10. Decrease in haemoglobin saturation (as measured by oximetry) from baseline value within past three months of > 10%<br>11. New finding (s) on chest radiograph
Rosenfeld definition (2001) (5)	≥ 6	The PEx score consists of six common clinical findings each weighted by a certain coefficient (in brackets):<br><br>For Model 1, score = 1.8 (exercise) + 1.5 (cough) + 1.5 (sputum/chest congestion) + 1.6 (absenteeism) + 1.2 (breath sounds) + 1.1 (appetite). The critical value (that value that maximizes sensitivity and specificity) is 2.6.<br><br>For Model 2, score = 1.7 (exercise) + 1.6 (cough) + 1.4 (sputum/chest congestion) + 1.7 (absenteeism) + 1.2 (breath sounds) + .9 (appetite) – 0.05 (change in FEV1). The critical value is 2.5.
Akron Pulmonary Eaxcerbation Score (PES) (6)	≥ 6	This score contains 14 elements that are divided into systemic, pulmonary signs and symptoms, and objective measurements. A score of five or above is required to meet the criteria for a pulmonary exacerbation. Further details of the PES elements are shown in Table 1.

Table 1. Akron Pulmonary Exacerbation Score

**Systemic signs/symptoms**	
Fever > 38 °C in the prior 2 weeks	Yes=1, No=0
Malaise or fatigue in the prior 2 weeks	Yes=1, No=0
Increased or new school absences in the prior 2 weeks	Yes=2, No=0
Anorexia or poor appetite in the prior 2 weeks	Yes=1, No=0
Weight loss (≥ 5%) of poor weight gain compared to last clinic visit (or in the last 3 months)	Yes=2, No=0
**Pulmonary signs/symptoms**	
Increased cough (frequency, intensity, duration) for ≥ 1 week	None=1, Mild=1, Significant=2
Major change in sputum or change in chest congestion for ≥ 1 week	None=1, Mild=1, Significant=2
Increased dyspnoea on exertion or shortness of breath	Yes=2, No=0
Change in chest exam or increased work of breathing or respiratory rate	Yes= 2, No=0
**Objective measurement**	
Decrease in FEV_1 (compared to highest value of the prior 6 months)	<10%=0, ≥10%=3, ≥15%=5
New chest radiograph abnormality	None=1, Increased air trapping or mucus plugging=1, New atelectasis or infiltrate=2, Pneumothorax=5
Haemoptysis	None=0, Streaked=3, Increased or new onset=5
Decreased oxygen saturation from baseline (compared to highest value of the prior 6 months)	<4% change=0, ≥4% decrease=2, ≥10% decrease=5

References:

1. Fuchs HJ, Borowitz DS, Christiansen DH, Morris EM, Nash ML, Ramsey BW, et al. Effect of aerosolized recombinant human dnase on exacerbations of respiratory symptoms and on pulmonary function in patients with cystic fibrosis. New England Journal of Medicine [Internet]. 1994; 331(10):[637–42 pp.]. Available from: https://doi.org/10.1056/NEJM199409083311003.

2. Bilton D, Canny G, Conway S, Dumcius S, Hjelte L, Proesmans M, et al. Pulmonary exacerbation: Towards a definition for use in clinical trials. Report from the EuroCareCF Working Group on outcome parameters in clinical trials. Journal of Cystic Fibrosis [Internet]. 2011; 10:[S79–S81 pp.]. Available from: https://www.scopus.com/inward/record.uri?eid=2-s2.0-79958077769&doi=10.1016%2fS1569-1993%2811%2960012-X&partnerID=40&md5=8579b763de430f6e5c1e617e776d2115.

3. Treggiari MM, Rosenfeld M, Mayer-Hamblett N, Retsch-Bogart G, Gibson RL, Williams J, et al. Early anti-pseudomonal acquisition in young patients with cystic fibrosis: Rationale and design of the EPIC clinical trial and observational study. Contemporary Clinical Trials [Internet]. 2009; 30(3):[256–68 pp.]. Available from: https://www.scopus.com/inward/record.uri?eid=2-s2.0-67349204479&doi=10.1016%2fj.cct.2009.01.003&partnerID=40&md5=c46c4332cb84ed06aff076bca92af613.

4. Cystic Fibrosis Foundation. Microbiology and infectious disease in cystic fibrosis. Cystic Fibrosis Foundation. 1994;V(I):25.

5. Rosenfeld M, Emerson J, Williams-Warren J, Pepe M, Smith A, Montgomery AB, Ramsey B. Defining a pulmonary exacerbation in cystic fibrosis. Journal of Pediatrics [Internet]. 2001 [cited RAYYAN-INCLUSION: {"Maryam"=>"Included"}; 139(3):[359–65 pp.]. Available from: http://ovidsp.ovid.com/ovidweb.cgi?T=JS&CSC=Y&NEWS=N&PAGE=fulltext&D=med4&AN=11562614 http://library.ncl.ac.uk/openurl/?sid=OVID&isbn=&issn=0022-3476&volume=139&issue=3&date=2001&title=Journal+of+Pediatrics&atitle=Defining+a+pulmonary+exacerbation+in+cystic+fibrosis.&aulast=Rosenfeld+M&spage=359.

6. Kraynack NC, McBride JT. Improving care at cystic fibrosis centers through quality improvement. Seminars in Respiratory and Critical Care Medicine [Internet]. 2009; 30(5):[547–58 pp.].
